# Supplementary material for: Contribution of the IGCR1 regulatory element and the 3′Igh CTCF-binding elements to regulation of Igh V(D)J recombination
Source: Proc Natl Acad Sci U S A. 2023 Jun 20;120(26):e2306564120. doi: 10.1073/pnas.2306564120 (PMC10293834; doi:10.1073/pnas.2306564120)
Supplement: Supplementary file 1 — Appendix 01 (PDF) [file pnas.2306564120.sapp.pdf]

Supplementary Information for

Contribution of the IGCR1 regulatory element and the 3′*Igh* CTCF-binding elements to regulation of *Igh* V(D)J recombination

Zhuoyi Liang[a,b,c,1,2], Lijuan Zhao[a,b,c,1], Adam Yongxin Ye[a,b,c,1], Sherry G. Lin[a,b,c,1], Yiwen Zhang[a,b,c], Chunguang Guo[a,b,c], Hai-Qiang Dai[a,b,c,3], Zhaoqing Ba[a,b,c,2,4], Frederick W. Alt[a,b,c,2]

[a]HHMI, Boston Children's Hospital, Boston, MA 02115; [b]Program in Cellular and Molecular Medicine, Boston Children's Hospital, Harvard Medical School, Boston, MA 02115; [c]Department of Genetics, Harvard Medical School, Boston, MA 02115.

[1]Z.L., L.Z., A.Y.Y., and S.G.L. contributed equally to this work.

[2]To whom correspondence may be addressed. Email: zhuoyi.liang@childrens.harvard.edu; bazhaoqing@nibs.ac.cn; alt@enders.tch.harvard.edu.

[3]Present address: Center for Excellence in Molecular Cell Science, Chinese Academy of Sciences, Shanghai 200031, China.

[4]Present address: National Institute of Biological Sciences, #7 Science Park Rd, Beijing 102206, China.

This PDF file includes:

Figures S1 to S10

Tables S1 to S5

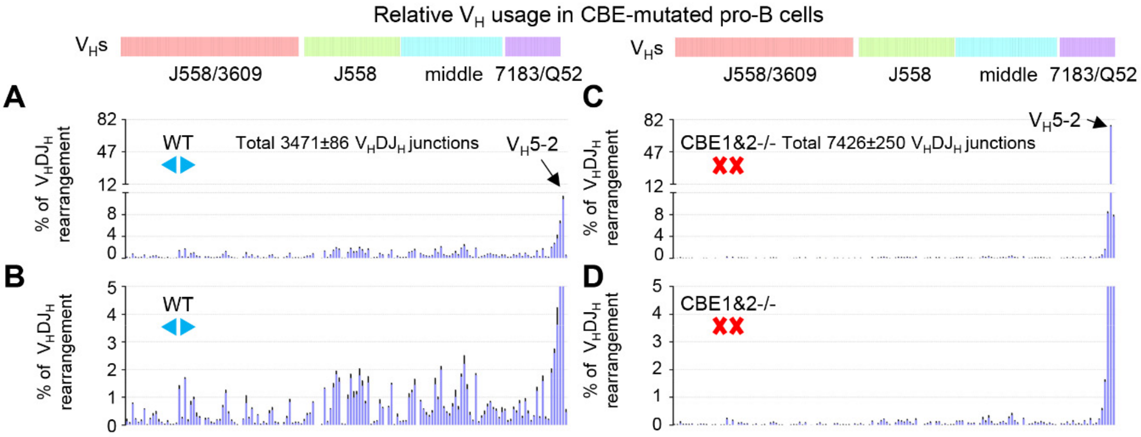

Figure S1. Relative V_H utilization in WT and CBE1&2$^{-/-}$ pro-B cells. (A-D) Relative percentage of V_Hs utilization normalized to the indicated V_HDJ_H junction number in WT (A, B) and CBE1&2$^{-/-}$ (C, D) pro-B cells (*n*=3 mice, mean±SEM; percentages are plotted from the data of Fig. 1B, D).

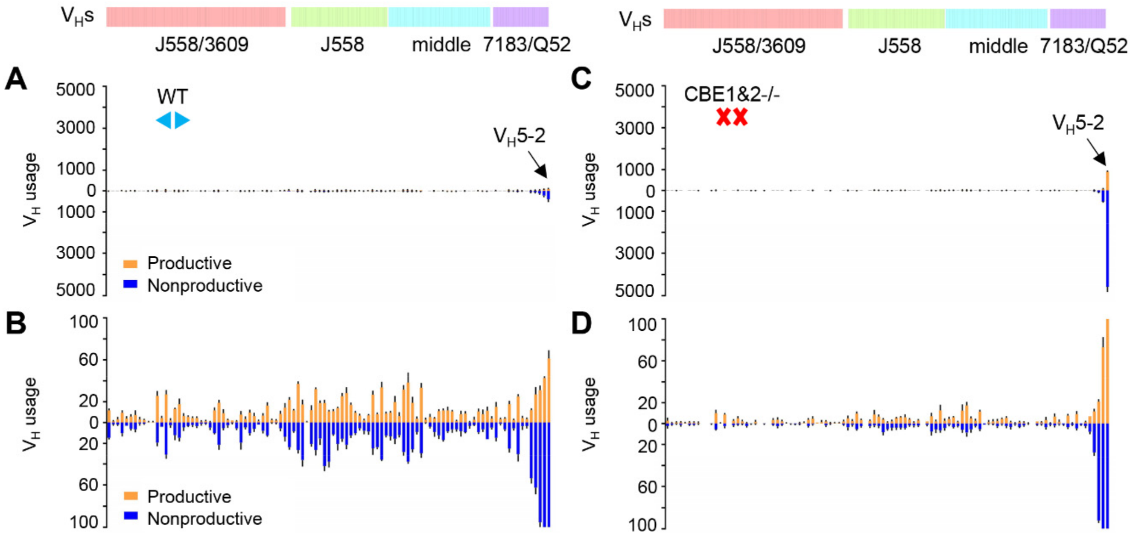

Figure S2. Productive and nonproductive rearrangements in WT and CBE1&2$^{-/-}$ pro-B cells. (A-D) Each panel shows the productive (orange) or nonproductive (blue) V_Hs usage in WT (A, B) and CBE1&2$^{-/-}$ (C, D) pro-B cells. Productive and nonproductive rearrangements are analyzed using a custom pipeline (*n*=3 mice, mean±SEM; see Methods and SI Appendix, Table S2).

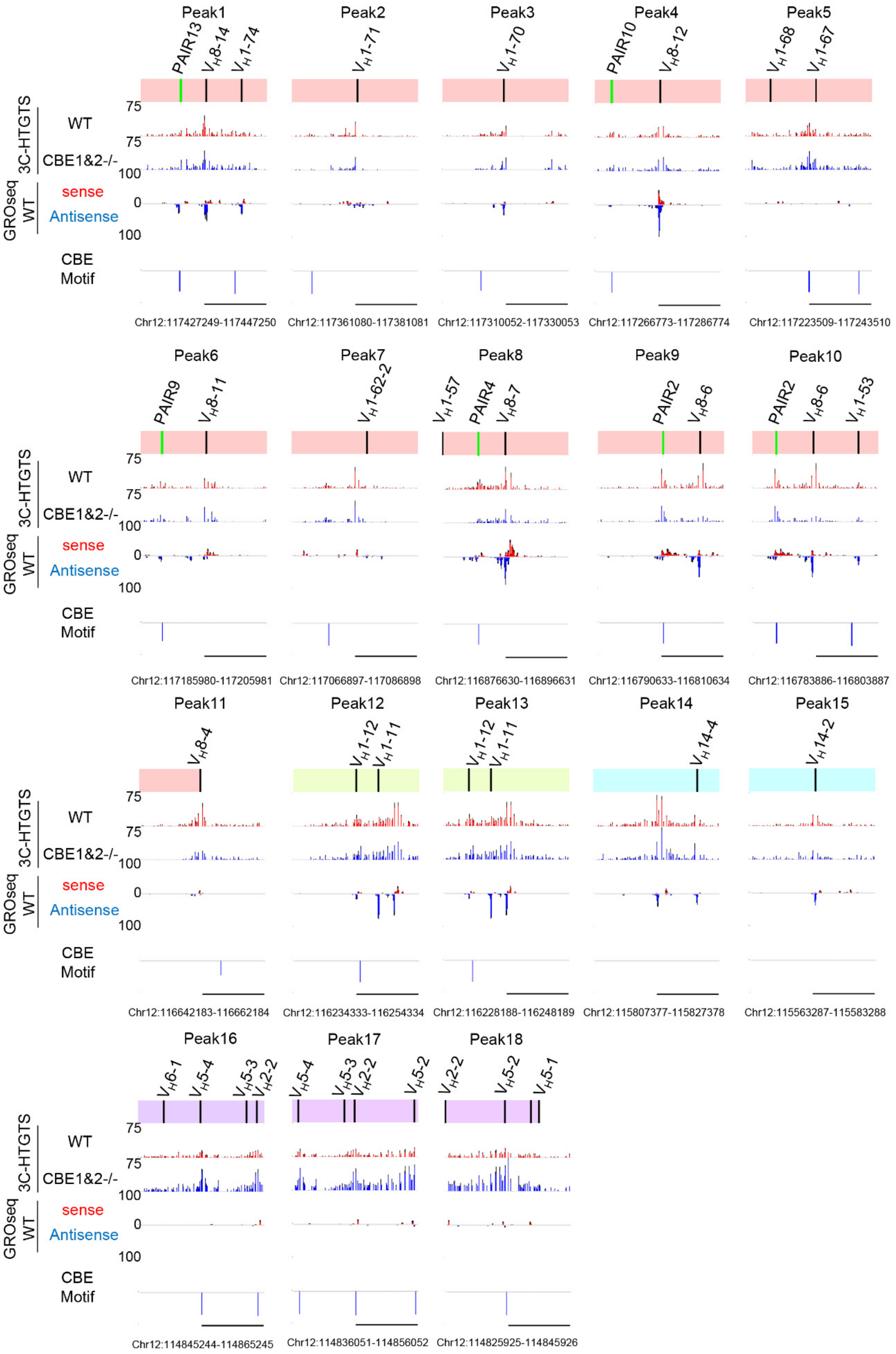

Peak1
Peak2
Peak3
Peak4
Peak5
PAIR13
VH8-14
VH1-74
VH1-71
VH1-70
PAIR10
VH8-12
VH1-68
VH1-67
3C-HTGTS
WT
CBE1&2-/-
GROseq WT
sense
Antisense
CBE Motif
75
100
0
Chr12:117427249-117447250
Chr12:117361080-117381081
Chr12:117310052-117330053
Chr12:117266773-117286774
Chr12:117223509-117243510
Peak6
Peak7
Peak8
Peak9
Peak10
PAIR9
VH8-11
VH1-62-2
VH1-57
PAIR4
VH8-7
PAIR2
VH8-6
VH1-53
Chr12:117185980-117205981
Chr12:117066897-117086898
Chr12:116876630-116896631
Chr12:116790633-116810634
Chr12:116783886-116803887
Peak11
Peak12
Peak13
Peak14
Peak15
VH8-4
VH1-12
VH1-11
VH14-4
VH14-2
Chr12:116642183-116662184
Chr12:116234333-116254334
Chr12:116228188-116248189
Chr12:115807377-115827378
Chr12:115563287-115583288
Peak16
Peak17
Peak18
VH6-1
VH5-4
VH5-3
VH2-2
VH5-2
VH5-1
Chr12:114845244-114865245
Chr12:114836051-114856052
Chr12:114825925-114845926

Figure S3. The major RC interactions, transcriptions, and CBE motifs in cultured RAG2-deficient primary pro-B cells. Zoom-in profiles of 3C-HTGTS, GRO-seq, CBE motif sites signals for ±10kb regions of 18 representative peaks in Fig. 2A from WT (red) and CBE1&2$^{-/-}$ (blue) cultured RAG2-deficient primary pro-B cells (*n*=3 mice, mean±SEM). PAIR elements (green bars) located in each peak are also shown above. Peaks 1-9, 12-15 are called in WT and CBE1&2$^{-/-}$; Peaks 10-11 are called only in WT; Peaks 16-18 are called only in CBE1&2$^{-/-}$.

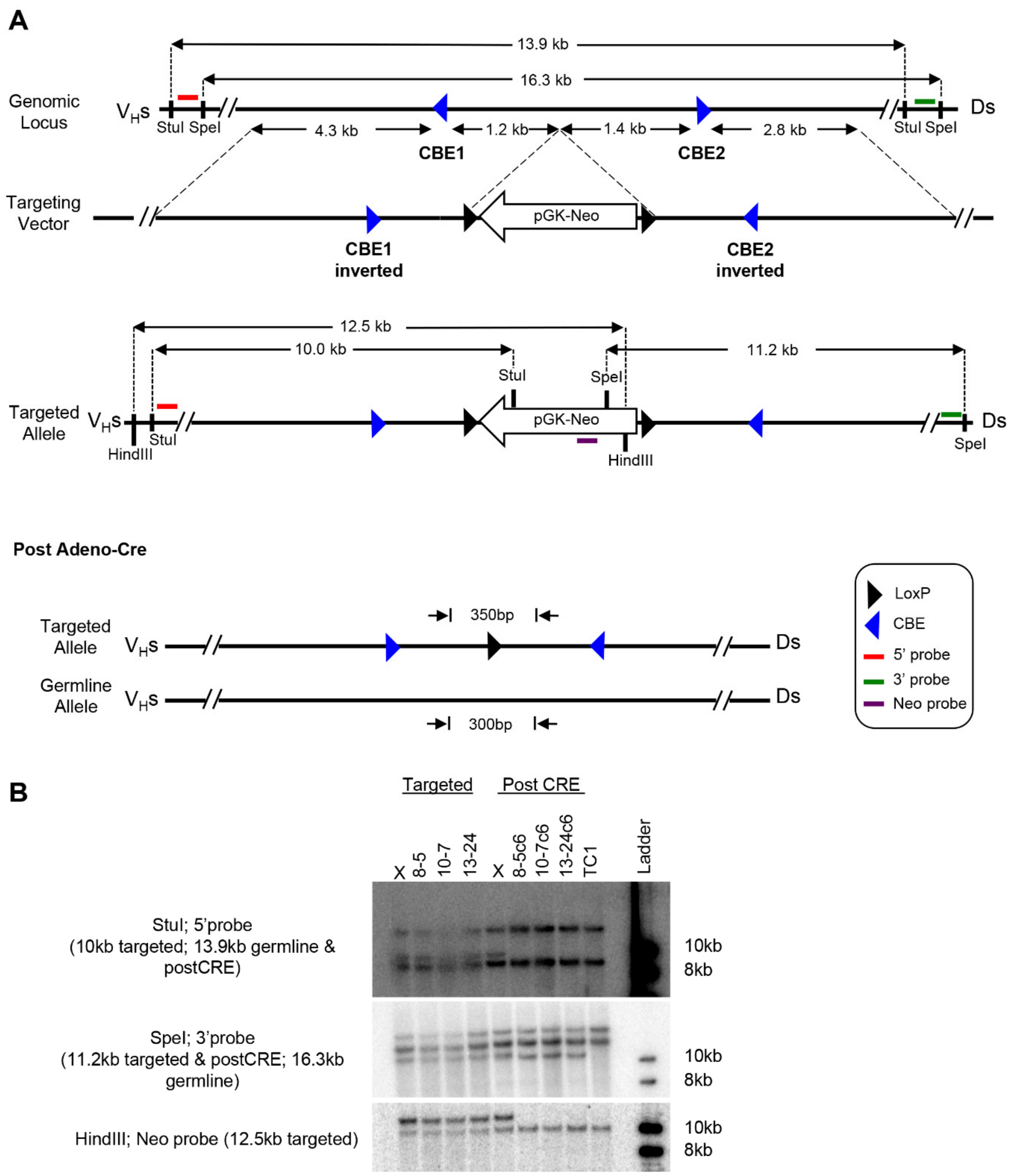

Figure S4. Generation and validation of CBE1 or CBE2 inversion mice. (A) Schematic diagram of the targeting strategy to generate CBE1 or CBE2 inversion in ES cells. Green and red lines indicate positions of probes used to confirm the insertion (see Methods). (B) Selection of inversion positive ES Cell clones by Southern blot (TC1: parental ES cell clone; 8-5c6, 10-7c6 and 13-24c6 indicate ES cell clones with insertion replacements after Adeno-Cre).

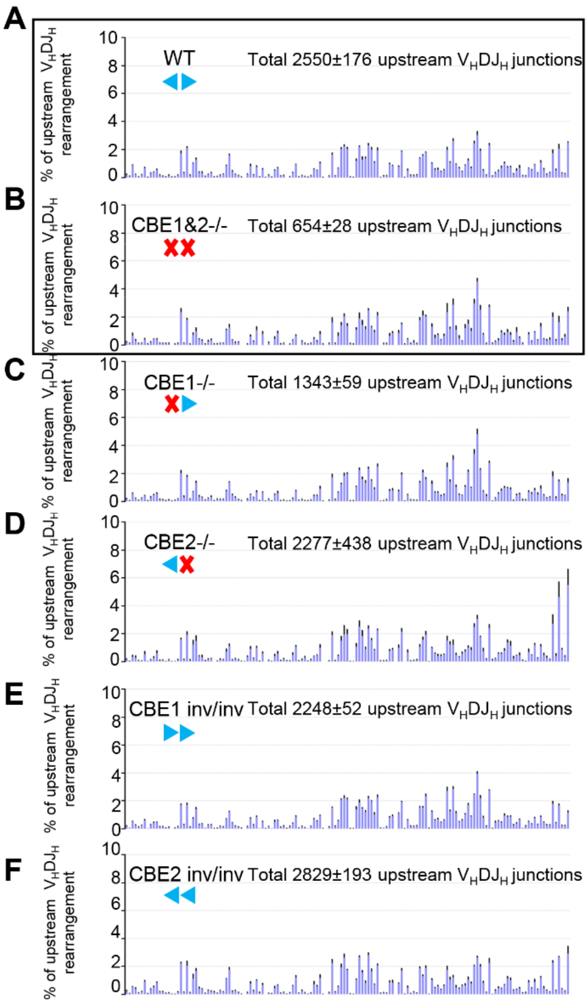

Figure S5 Relative V_H utilization in WT and indicated IGCR1/CBEs mutated pro-B cells. (A-F) Each panel shows the relative percentage of upstream V_Hs beyond the five most proximal V_Hs normalized to the indicated V_HDJ_H junction number. Upstream V_Hs junctions are extracted from the data of Fig. 1B, C and Fig.3 A-D. Panel A, B are reproduced from Fig. 1H, I (*n*=3 mice, mean±SEM).

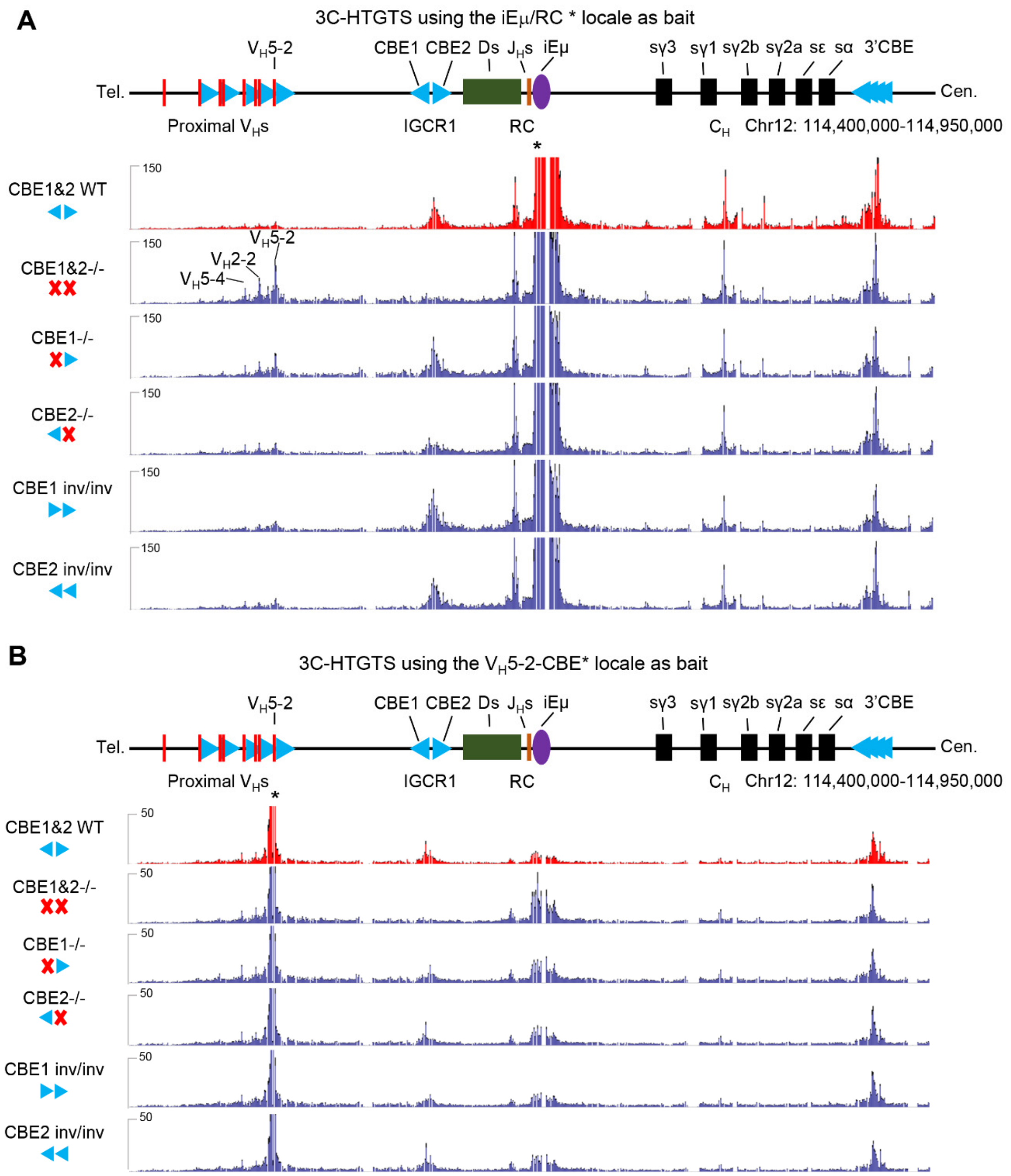

Figure S6. 3C-HTGTS profiles bating from RC and V_H5-2-CBE in IGCR1/WT and IGCR1/CBEs mutated *v-Abl* cells. (A) 3C-HTGTS signal counts of IGCR1/WT (red) and IGCR1/CBEs mutated (blue) RAG2-deificient *v-Abl* lines baiting from iEμ/RC (*). Each library was normalized to 70,566 total junctions (*n*=6 repeats from 2 independent clones, mean±SEM). (B) 3C-HTGTS signal counts of IGCR1/WT (red) and IGCR1/CBEs mutated (blue) RAG2-deificient *v-Abl* lines baiting from V_H5-2-CBE (*). Each library was normalized to 14,018 total junctions (*n*=6 repeats from 2 independent clones, mean±SEM).

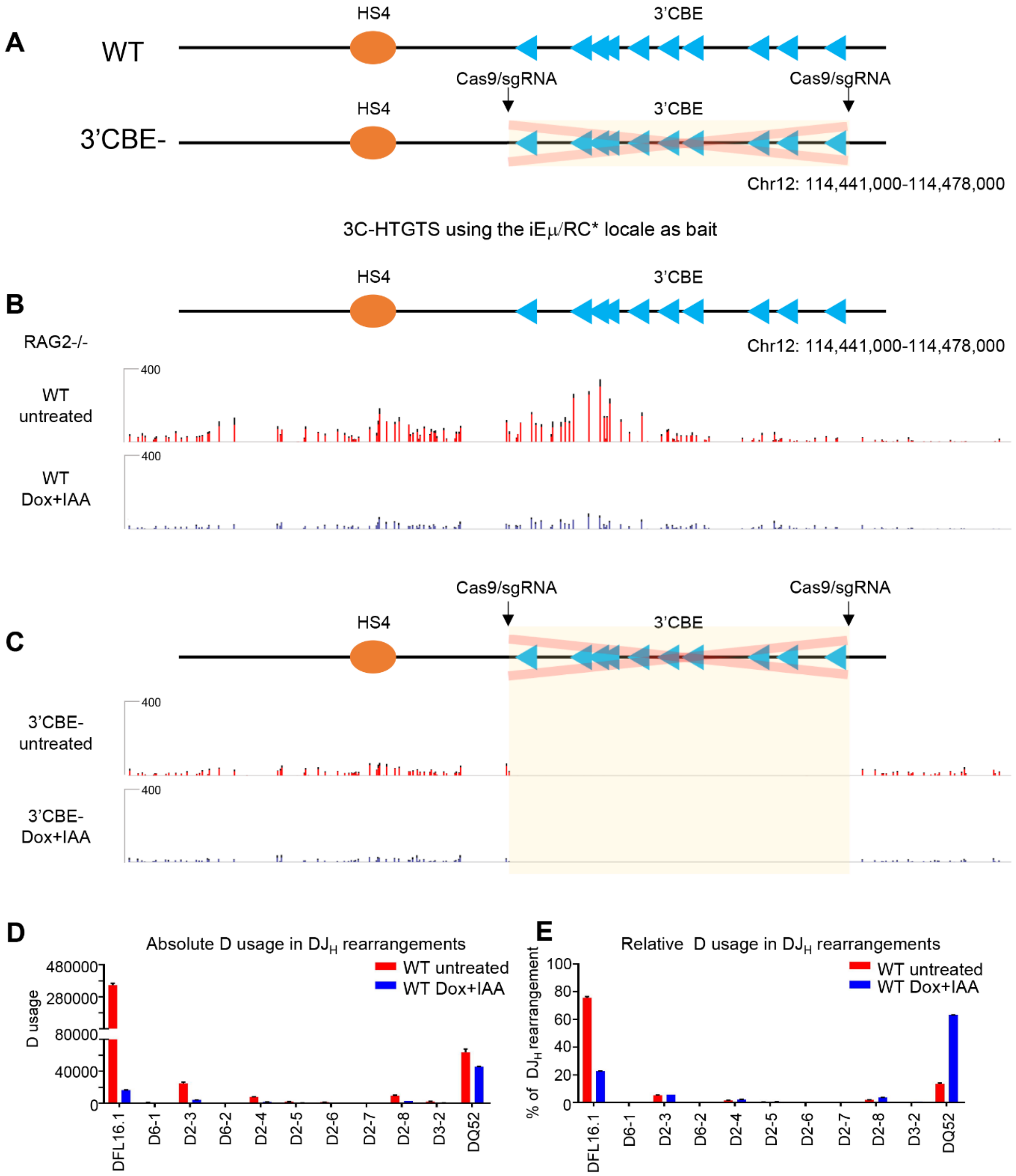

Figure S7. Role of 3'*Igh* CBEs in RC activity during loop extrusion. (A) Generation of 3'CBE⁻ by Cas9/sgRNAs in single *Igh* WAPL-degron *v-Abl* lines. (B-C) Zoom-in 3C-HTGTS profiles of 3'*Igh* CBE in WT (B) and 3'CBE⁻ (C) WAPL-degron *v-Abl* cells with or without Dox/IAA treatments (Chr12: 114,441,000-114,478,000; see Fig. 5 B). (D, E) Absolute (D) and relative (E) D usage in D to J_H1-4 rearrangements in untreated and WAPL-depleted WT *v-Abl* cells. HTGTS-V(D)J-Seq data was extracted from a prior study in which employed primers

specific to each of the four mouse J_Hs (22: GSM4593296-GSM4593303). HTGTS-V(D)J-Seq libraries were generated using combined J_H1-4 primers and relative percentage was normalized to 465,530 DJ_H absolute junctions in untreated or 72,283 DJ_H absolute junctions in WAPL-depleted cells. The general trends for absolute and relative D usage based on utilizing combined primers for all four J_Hs are similar to our findings based on utilization J_H4 primer (Fig. 4 M, N). The absolute usage data for all four J_Hs in these experiments is shown in Table S4.

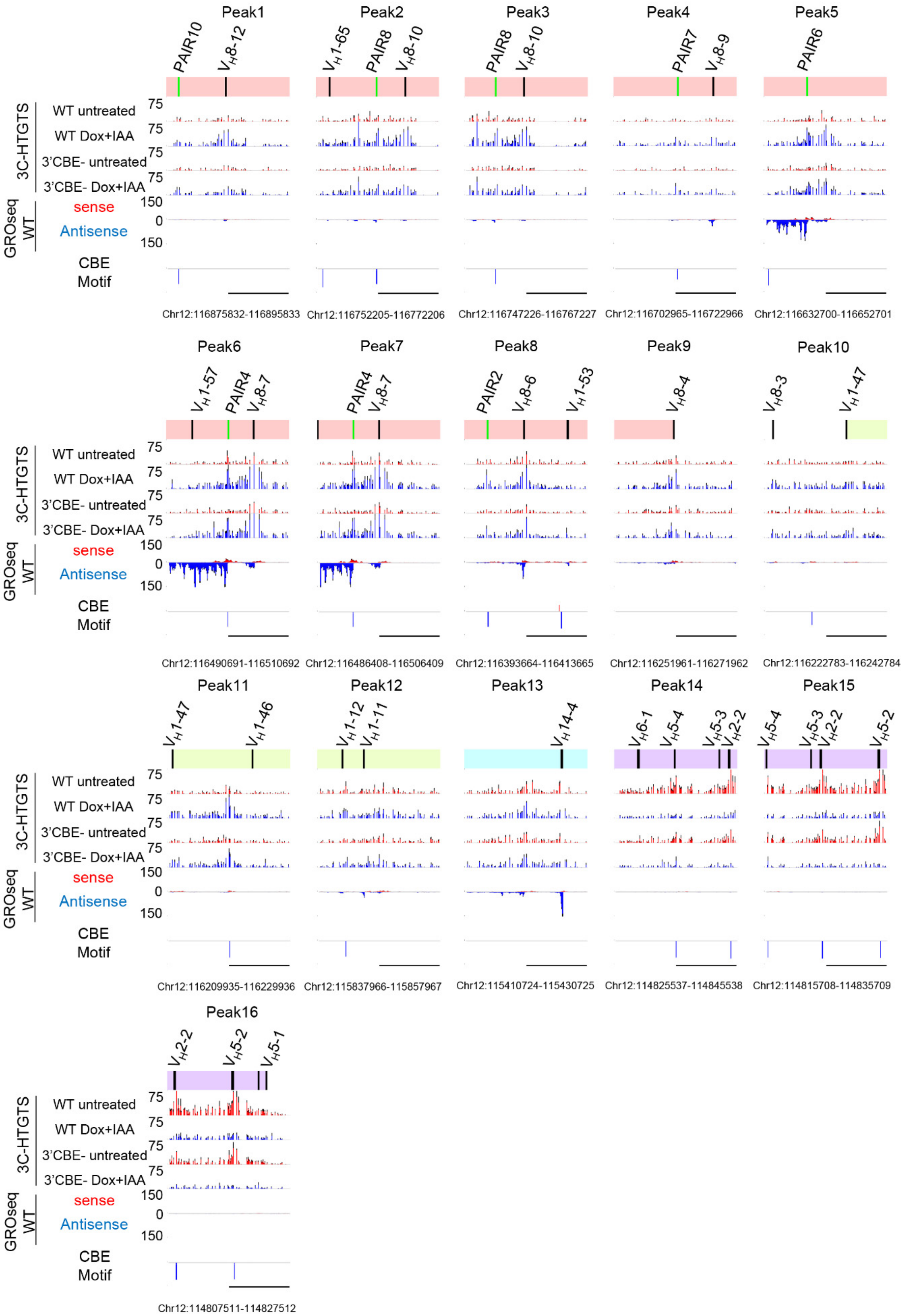

Peak1
PAIR10
VH8-12
Peak2
VH1-65
PAIR8
VH8-10
Peak3
PAIR8
VH8-10
Peak4
PAIR7
VH8-9
Peak5
PAIR6
3C-HTGTS
WT untreated
WT Dox+IAA
3'CBE- untreated
3'CBE- Dox+IAA
GROseq WT
sense
Antisense
CBE Motif
75
150
0
Chr12:116875832-116895833
Chr12:116752205-116772206
Chr12:116747226-116767227
Chr12:116702965-116722966
Chr12:116632700-116652701
Peak6
VH1-57
PAIR4
VH8-7
Peak7
PAIR4
VH8-7
Peak8
PAIR2
VH8-6
VH1-53
Peak9
VH8-4
Peak10
VH8-3
VH1-47
Chr12:116490691-116510692
Chr12:116486408-116506409
Chr12:116393664-116413665
Chr12:116251961-116271962
Chr12:116222783-116242784
Peak11
VH1-47
VH1-46
Peak12
VH1-12
VH1-11
Peak13
VH14-4
Peak14
VH6-1
VH5-4
VH5-3
VH2-2
Peak15
VH5-4
VH5-3
VH2-2
VH5-2
Chr12:116209935-116229936
Chr12:115837966-115857967
Chr12:115410724-115430725
Chr12:114825537-114845538
Chr12:114815708-114835709
Peak16
VH2-2
VH5-2
VH5-1
Chr12:114807511-114827512

Figure S8. The major RC interactions, transcriptions, and CBE motifs in WT and 3'CBE^{-} WAPL-depleted *v-Abl* cells. Zoom-in profiles of 3C-HTGTS, GRO-seq, CBE motif sites signals for ±10kb regions of 16 representative peaks in Fig. 5A from WT and 3'CBE^{-} WAPL-depleted *v-Abl* cells (*n*=3 repeats from 3 independent clones, mean±SEM). PAIR elements (green bars) located in each peak are also shown above. Peaks 1, 6-9, 12-13 are shared in WAPL-depleted WT *v-Abl* cells and WT pro-B cells (SI Appendix, Fig. S3); Peaks 2-5, 10-11 are called in WAPL-depleted *v-Abl* cells; Peak1 is called only in WAPL-depleted WT *v-Abl* cells; Peaks 14-16 are only present in untreated WT and 3'CBE^{-} *v-Abl* lines. The reference GRO-seq sequencing data was extracted from 22: GSM4593308-GSM4593311.

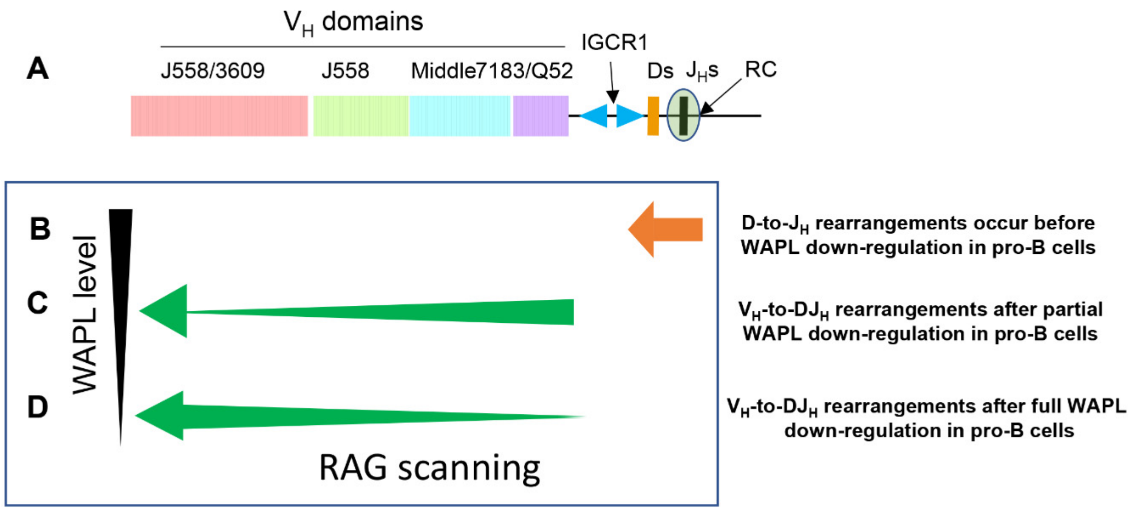

Figure S9. Gradual WAPL down-regulation regulates ordered V(D)J recombination in developing progenitor B cells. (A) Schematic of the murine *Igh* locus illustrating V_Hs, Ds, J_Hs, the J_H-based RC and IGCR1 (not to scale). (B) Robust D-to-J_H rearrangements occur before the WAPL down-regulation in the absence of V_H-to-DJ_H rearrangements due to RAG scanning of upstream chromatin being impeded by IGCR1. (C) Once WAPL down-regulation reaches sufficient levels, IGCR1 on proximal V_H CBEs are partially neutralized allowing scanning from a new DJ_H RC (not illustrated) to enter the V_H domain, but rearrangements are focused mostly on proximal V_Hs due to incomplete neutralization of their CBEs. Proximal V_HsRSS-associated rearrangements largely obviate scanning to upstream V_Hs. (D) Complete physiological down-regulation of WAPL neutralizes IGCR1 and V_HRSS-associated CBEs, preventing dominant proximal V_H rearrangements, and promotes RAG scanning and V_H utilization across the entire V_Hs locus (For additional details see text).

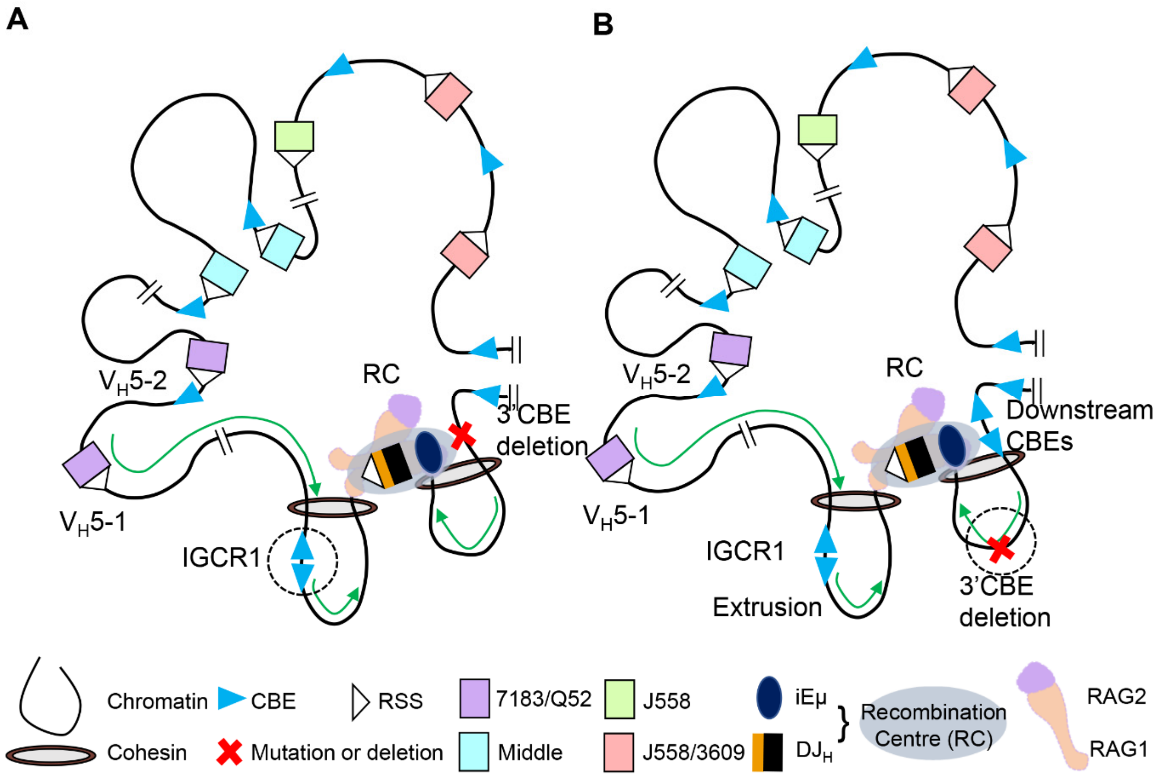

Figure S10. Roles of IGCR1/CBEs and 3'*Igh* CBEs in *Igh* loop-extrusion mediated RAG scanning. (A) 3'*Igh* CBEs reinforce the impediment activity of RC and promote the optimal RAG scanning. (B) Downstream CBEs appear to gain robust interactions with the RC upon the deletion of 3'*Igh* CBEs.

Supplementary Table 1 | Absolute utilization of Igh VH and D segments in WT and IGCR1/CBEs mutated pro-B cells.

		WT (n=3)					CBE1&2 -/- (n=3)					CBE1 -/- (n=3)					CBE2 -/- (n=3)					CBE1 inv/inv (n=3)					CBE2 inv/inv (n=3)				
**VH domains**		#1	#2	#3	Average	s.d.	#1	#2	#3	Average	s.d.	#1	#2	#3	Average	s.d.	#1	#2	#3	Average	s.d.	#1	#2	#3	Average	s.d.	#1	#2	#3	Average	s.d.
	Ighv1-86P	9	5	0	5	5	2	0	1	1	1	4	2	3	3	1	7	6	0	4	4	4	9	1	5	4	2	6	3	4	2
	Ighv1-85	4	2	0	2	2	1	0	2	1	1	1	1	2	1	1	1	1	0	1	1	0	1	4	2	2	0	3	2	2	2
	Ighv1-84	24	30	25	26	3	2	0	13	5	7	5	8	7	7	2	22	5	5	11	10	11	16	13	13	3	12	20	6	13	7
	Ighv1-83P	8	8	5	7	2	3	1	2	2	1	6	3	2	4	2	14	10	1	8	7	0	8	4	4	4	11	17	6	11	6
	Ighv1-82	2	4	3	3	1	1	3	1	2	1	3	3	0	2	2	6	0	2	3	3	5	3	0	3	3	5	4	2	4	2
	Ighv1-81	3	4	8	5	3	3	0	1	1	2	1	3	2	2	1	3	0	0	1	2	5	6	3	5	2	6	5	5	5	1
	Ighv1-80	18	22	19	20	2	2	4	2	3	1	9	5	5	6	2	34	5	4	14	17	16	12	15	14	2	18	33	19	23	8
	Ighv1-79P	0	1	0	0	1	0	0	0	0	0	0	0	0	0	0	1	0	0	0	1	1	2	0	1	1	2	1	0	1	1
	Ighv1-78	10	6	5	7	3	1	0	1	1	1	2	6	2	3	2	1	2	2	2	1	7	5	10	7	3	13	14	10	12	2
	Ighv1-77	16	8	11	12	4	2	3	3	3	1	3	6	5	5	2	11	5	2	6	5	5	8	19	11	7	19	16	7	14	6
	Ighv1-76	10	21	11	14	6	4	1	1	2	2	9	8	3	7	3	24	20	4	16	11	11	7	16	11	5	23	27	15	22	6
	Ighv1-75	9	1	3	4	4	1	0	2	1	1	3	3	0	2	2	5	3	0	3	3	3	5	4	4	1	3	6	2	4	2
	Ighv1-74	3	4	3	3	1	0	0	0	0	0	4	2	4	3	1	3	3	0	2	2	2	7	6	5	3	1	10	5	5	5
	Ighv1-73P	0	0	2	1	1	0	1	0	0	1	1	1	1	1	0	0	0	0	0	0	0	1	1	1	1	2	2	1	2	1
	Ighv8-13	4	0	7	4	4	0	0	2	1	1	1	3	1	2	1	3	3	2	3	1	0	0	1	0	1	1	4	3	3	2
	Ighv1-72	1	1	0	1	1	0	0	0	0	0	0	0	1	0	1	0	0	0	0	0	1	1	0	1	1	0	2	0	1	1
	Ighv1-71	1	1	0	1	1	0	0	1	0	1	1	0	1	1	1	0	0	0	0	0	0	3	1	1	2	0	3	0	1	2
	Ighv1-70P	2	3	1	2	1	1	0	1	1	1	0	2	4	2	2	4	4	1	3	2	4	2	3	3	1	7	6	5	6	1
	Ighv8-12	42	40	53	45	7	14	12	21	16	5	20	26	22	23	3	65	35	22	41	22	25	30	32	29	4	49	66	48	54	10
	Ighv1-69	6	11	8	8	3	1	0	0	0	1	4	5	4	4	1	2	4	2	3	1	6	18	9	11	6	3	9	1	4	4
**J558/3609**	Ighv1-67	58	57	58	58	1	10	15	9	11	3	24	26	16	22	5	64	32	41	46	17	41	25	36	34	8	38	81	52	57	22
	Ighv1-66	7	6	1	5	3	0	0	0	0	0	6	0	2	3	3	3	4	0	2	2	2	2	2	2	0	2	2	3	2	1
	Ighv8-11	19	23	34	25	8	7	1	5	4	3	12	12	11	12	1	67	19	14	33	29	13	22	14	16	5	20	31	36	29	8
	Ighv1-64	28	39	30	32	6	8	5	5	6	2	15	19	21	18	3	75	20	23	39	31	35	38	40	38	3	46	74	22	47	26
	Ighv1-63	4	20	9	11	8	2	4	2	3	1	2	4	3	3	1	16	10	5	10	6	4	7	3	5	2	7	23	8	13	9
	Ighv1-62-3	7	9	12	9	3	2	3	4	3	1	7	4	4	5	2	10	1	5	5	5	6	6	4	5	1	10	8	7	8	2
	Ighv1-62P	0	0	0	0	0	0	0	0	0	0	0	0	0	0	0	0	0	0	0	0	0	0	0	0	0	0	0	0	0	0
	Ighv1-61	10	7	3	7	4	0	1	0	0	1	5	4	1	3	2	7	2	2	4	3	3	6	8	6	3	3	10	4	6	4
	Ighv1-59	9	8	8	8	1	0	2	4	2	2	5	1	2	3	2	5	5	2	4	2	2	8	10	7	4	6	11	4	7	4
	Ighv1-58	5	0	2	2	3	0	0	0	0	0	0	1	2	1	1	0	0	0	0	0	2	0	1	1	1	2	2	5	3	2
	Ighv8-8	2	3	2	2	1	1	0	0	0	1	2	1	2	2	1	0	0	0	0	0	0	1	2	1	1	1	1	1	1	0
	Ighv8-7P	6	6	7	6	1	2	1	0	1	1	1	1	1	1	0	11	1	2	5	6	4	2	4	3	1	7	4	6	6	2
	Ighv1-56	4	9	4	6	3	0	0	0	0	0	2	1	3	2	1	7	2	2	4	3	2	3	1	2	1	2	3	4	3	1
	Ighv1-55	26	20	20	22	3	2	7	5	5	3	10	11	6	9	3	40	13	6	20	18	15	19	15	16	2	22	37	21	27	9
	Ighv1-54	35	49	37	40	8	3	9	7	6	3	17	23	23	21	3	42	18	22	27	13	34	33	28	32	3	47	49	34	43	8
	Ighv1-53	11	13	19	14	4	1	5	4	3	2	6	8	2	5	3	5	2	2	3	2	13	14	6	11	4	9	2	2	4	4
	Ighv1-52	4	8	4	5	2	0	1	0	0	1	5	3	3	4	1	5	7	4	5	2	4	5	3	4	1	6	8	9	8	2
	Ighv1-50	3	3	2	3	1	0	1	0	0	1	0	3	1	1	2	5	2	2	3	2	0	2	7	3	4	5	4	3	4	1
	Ighv8-5	1	0	0	0	1	0	0	0	0	0	1	0	2	1	1	2	0	0	1	1	0	2	2	1	1	1	1	0	1	1
	Ighv1-49	0	0	0	0	0	0	0	0	0	0	0	0	0	0	0	0	0	0	0	0	0	0	0	0	0	0	0	0	0	0
	Ighv8-4	7	6	8	7	1	0	1	1	1	1	1	2	4	2	2	3	8	5	5	3	2	7	5	5	3	5	0	2	2	3
	Ighv1-47	44	18	19	27	15	2	1	1	1	1	3	7	11	7	4	21	16	20	19	3	11	11	27	16	9	27	12	9	16	10
	Ighv1-43	8	11	6	8	3	2	0	0	1	1	5	5	2	4	2	9	7	2	6	4	6	7	8	7	1	2	12	4	6	5
	Ighv1-42	22	19	19	20	2	5	4	9	6	3	7	15	9	10	4	29	25	16	23	7	15	26	29	23	7	20	35	18	24	9
	Ighv1-40P	3	1	0	1	2	0	1	0	0	1	2	1	1	1	1	1	0	1	1	1	0	3	0	1	2	3	5	0	3	3
	Ighv1-39	13	13	11	12	1	4	8	4	5	2	8	8	6	7	1	17	18	7	14	6	13	10	10	11	2	22	20	6	16	9
	Ighv1-38P	0	2	0	1	1	0	0	0	0	0	2	1	0	1	1	0	0	0	0	0	1	0	0	0	1	0	0	0	0	0
	Ighv1-37	5	2	3	3	2	1	1	1	1	0	2	4	2	3	1	4	1	2	2	2	4	5	2	4	2	5	4	1	3	2
	Ighv1-36	0	0	3	1	2	0	0	0	0	0	1	0	0	0	1	0	1	1	1	1	1	1	0	1	1	2	1	0	1	1
	Ighv1-34	13	10	21	15	6	0	4	0	1	2	7	7	4	6	2	23	3	5	10	11	13	20	13	15	4	9	20	2	10	9
	Ighv1-33P	23	20	13	19	5	4	8	3	5	3	10	3	7	7	4	29	15	4	16	13	13	18	17	16	3	27	24	12	21	8
	Ighv1-32P	3	5	1	3	2	1	1	0	1	1	0	2	4	2	2	2	3	0	2	2	4	3	3	3	1	4	4	3	4	1
	Ighv1-31	4	1	0	2	2	2	1	0	1	1	2	2	2	2	0	4	7	1	4	3	3	6	1	3	3	4	4	2	3	1
	Ighv1-30P	0	0	0	0	0	0	0	0	0	0	2	0	0	1	1	2	0	3	2	2	1	2	2	2	1	0	0	1	0	1
	Ighv1-28P	4	1	1	2	2	0	0	0	0	0	2	0	0	1	1	0	2	1	1	1	1	1	6	3	3	2	5	3	3	2
	Ighv1-27P	5	13	9	9	4	2	5	0	2	3	2	1	1	1	1	15	7	12	11	4	8	12	8	9	2	14	22	7	14	8
	Ighv1-26	34	27	25	29	5	5	3	3	4	1	8	10	12	10	2	45	21	14	27	16	19	23	19	20	2	31	40	19	30	11
	Ighv1-25P	2	3	1	2	1	0	1	0	0	1	0	0	2	1	1	3	2	2	2	1	0	1	3	1	2	2	0	2	1	1
	Ighv1-23	2	5	1	3	2	0	2	1	1	1	4	1	0	2	2	2	6	0	3	3	8	9	1	6	4	2	12	3	6	6
	Ighv1-22	3	4	1	3	2	1	1	0	1	1	0	1	1	1	1	2	10	1	4	5	0	1	1	1	1	5	3	3	4	1
	Ighv1-21P	1	1	2	1	1	0	2	0	1	1	1	0	0	0	1	3	1	0	1	2	3	2	1	2	1	1	1	0	1	1
**J558**	Ighv1-21-1P	2	9	5	5	4	0	0	0	0	0	2	0	2	1	1	8	4	1	4	4	8	3	3	5	3	1	3	0	1	2
	Ighv1-20	8	15	15	13	4	3	1	4	3	2	4	8	3	5	3	14	9	4	9	5	18	22	13	18	5	16	44	10	23	18
	Ighv1-19	15	15	12	14	2	1	1	0	1	1	9	2	3	5	4	21	11	8	13	7	10	15	12	12	3	10	22	15	16	6
	Ighv1-18	29	28	27	28	1	3	5	1	3	2	14	12	4	10	5	52	22	11	28	21	30	15	21	22	8	19	47	32	33	14
	Ighv1-17P	0	0	0	0	0	0	0	1	0	1	0	0	0	0	0	0	0	0	0	0	0	0	0	0	0	0	2	1	1	1
	Ighv1-16	0	0	0	0	0	0	0	0	0	0	0	0	0	0	0	0	0	0	0	0	0	0	0	0	0	0	0	0	0	0
	Ighv1-15	0	1	1	1	1	1	1	0	1	1	1	2	1	1	1	5	0	1	2	3	0	2	2	1	1	3	7	2	4	3
	Ighv1-14	45	47	44	45	2	5	10	11	9	3	23	21	34	26	7	46	37	40	41	5	23	30	54	36	16	53	87	49	63	21
	Ighv1-13P	3	6	2	4	2	0	0	0	0	0	4	2	1	2	2	4	0	2	2	2	4	0	2	2	2	4	12	3	6	5
	Ighv1-12	20	20	20	20	0	3	3	5	4	1	6	10	8	8	2	14	7	8	10	4	7	9	14	10	4	10	15	10	12	3
	Ighv1-11	41	43	57	47	9	7	11	13	10	3	23	10	16	16	7	64	21	17	34	26	55	54	40	50	8	51	54	41	49	7
	Ighv1-9	66	69	56	64	7	17	16	12	15	3	20	24	26	23	3	91	17	32	47	39	36	63	65	55	16	67	77	44	63	17
	Ighv15-2	59	59	44	54	9	6	9	4	6	3	30	24	28	27	3	76	51	29	52	24	35	50	45	43	8	44	56	30	43	13
	Ighv1-8P	2	1	1	1	1	0	0	2	1	1	1	0	1	1	1	0	0	0	0	0	1	1	1	1	0	2	4	1	2	2
	Ighv1-7	1	1	1	1	0	0	0	0	0	0	0	0	1	0	1	0	0	0	0	0	0	0	2	1	1	1	0	0	0	1
	Ighv10-3	40	32	26	33	7	5	5	6	5	1	15	10	19	15	5	35	14	22	24	11	21	19	39	26	11	27	43	18	29	13
	Ighv1-5	60	50	65	58	8	7	8	16	10	5	33	33	28	31	3	106	37	46	63	38	45	37	49	44	6	62	84	52	66	16
	Ighv1-4	47	29	26	34	11	10	4	13	9	5	14	23	22	20	5	75	46	16	46	30	25	40	31	32	8	30	53	34	39	12
	Ighv10-1	41	33	41	38	5	10	6	5	7	3	10	18	16	15	4	26	25	11	21	8	25	24	31	27	4	33	31	5	23	16
	Ighv1-2P	75	54	57	62	11	8	15	14	12	4	29	32	28	30	2	84	43	45	57	23	47	56	41	48	8	69	86	49	68	19
	Ighv8-2	56	53	38	49	10	6	4	9	6	3	24	18	19	20	3	49	32	25	35	12	43	53	48	48	5	30	65	35	43	19
	IGHV6-7	31	25	25	27	3	2	1	4	2	2	13	10	7	10	3	23	20	12	18	6	29	18	19	22	6	18	20	15	18	3
	IGHV6-6	63	41	54	53	11	21	9	12	14	6	43	24	23	30	11	87	44	42	58	25	47	39	49	45	5	69	76	40	62	19
	IGHV6-5	1	1	0	1	1	0	0	0	0	0	0	1	0	0	1	2	1	0	1	1	1	1	0	1	1	2	2	0	1	1
	IGHV6-4	6	1	1	3	3	0	0	0	0	0	0	0	0	0	0	3	1	0	1	2	4	2	4	3	1	4	5	1	3	2
	IGHV6-3	4	6	5	5	1	2	2	0	1	1	2	3	2	2	1	5	0	4	3	3	2	5	4	4	2	1	4	0	2	2
	IGHV12-3	30	18	32	27	8	1	5	4	3	2	13	11	7	10	3	14	16	17	16	2	13	18	16	16	3	21	22	13	19	5
	IGHV13-2	19	22	23	21	2	2	4	11	6	5	10	6	12	9	3	27	23	16	22	6	17	18	30	22	7	9	19	18	15	6
	IGHV3-7P	0	0	0	0	0	0	0	0	0	0	0	0	0	0	0	0	0	1	0	1	0	0	0	0	0	0	0	0	0	0
	IGHV9-4	23	21	23	22	1	5	1	12	6	6	5	10	11	9	3	36	14	23	24	11	20	33	33	29	8	21	20	13	18	4
	IGHV3-6	47	51	54	51	4	7	10	7	8	2	15	27	21	21	6	73	50	23	49	25	41	63	38	47	14	32	45	40	39	7
	IGHV13-1P	0	1	0	0	1	0	0	1	0	1	0	1	0	0	1	0	0	0	0	0	0	2	0	1	1	0	0	1	0	1
	IGHV3-5	15	12	4	10	6	4	1	3	3	2	9	4	3	5	3	14	22	9	15	7	15	25	8	16	9	14	6	7	9	4
	IGHV3-4	5	4	3	4	1	1	0	0	0	1	1	3	0	1	2	1	2	1	1	1	6	7	2	5	3	1	0	3	1	2
	IGHV7-4	6	5	1	4	3	1	2	0	1	1	3	0	0	1	2	5	1	2	3	2	2	2	6	3	2	6	2	3	4	2
	IGHV3-3P	3	7	4	5	2	0	1	2	1	1	1	0	1	1	1	0	4	1	2	2	8	2	3	4	3	0	4	2	2	2
	IGHV14-4	24	41	39	35	9	8	12	8	9	2	18	17	19	18	1	24	29	10	21	10	34	47	33	38	8	34	62	45	47	14
**Middle**	IGHV7-3	34	46	43	41	6	11	11	8	10	2	28	23	26	26	3	67	25	24	39	25	38	30	37	35	4	37	47	20	35	14
	IGHV9-2-1	48	65	38	50	14	13	10	8	10	3	23	21	20	21	2	64	31	40	45	17	56	56	43	52	8	55	58	32	48	14
	IGHV12-2-1	0	0	0	0	0	0	0	0	0	0	0	0	1	0	1	1	0	0	0	1	2	0	0	1	1	0	0	0	0	0
	IGHV9-3-1	31	28	29	29	2	6	6	5	6	1	20	24	19	21	3	30	23	14	22	8	20	22	31	24	6	25	45	14	28	16
	IGHV12-1-1	20	20	19	20	1	3	5	1	3	2	13	15	5	11	5	17	14	7	13	5	13	16	11	13	3	19	16	8	14	6
	IGHV9-3	17	8	13	13	5	6	5	4	5	1	10	3	9	7	4	15	4	12	10	6	14	17	20	17	3	6	22	9	12	9
	IGHV9-2	12	7	12	10	3	0	0	0	0	0	2	9	2	4	4	6	12	11	10	3	12	7	8	9	3	12	9	5	9	4
	IGHV9-1	11	16	20	16	5	4	2	4	3	1	5	4	4	4	1	20	11	4	12	8	12	15	12	13	2	15	21	10	15	6
	IGHV14-3	38	68	48	51	15	8	26	10	15	10	38	41	19	33	12	39	45	13	32	17	45	88	62	65	22	51	61	18	43	23

	IGHV11-2	36	31	29	32	4	6	2	15	8	7	13	23	26	21	7	28	36	18	27	9	28	24	15	22	7	25	30	31	29	3
	IGHV3-2	60	72	77	70	9	11	30	17	19	10	37	48	33	39	8	62	12	22	32	26	59	84	84	76	14	42	40	21	34	12
	IGHV4-2	21	19	24	21	3	14	3	4	7	6	13	21	15	16	4	17	14	5	12	6	13	29	28	23	9	18	19	5	14	8
	IGHV14-2P	13	15	18	15	3	3	1	2	2	1	11	6	4	7	4	12	7	7	9	3	12	12	10	11	1	17	16	5	13	7
	IGHV11-1	11	8	16	12	4	3	2	0	2	2	3	6	5	5	2	8	18	6	11	6	12	16	12	13	2	16	26	11	18	8
	IGHV3-1	29	19	21	23	5	4	9	4	6	3	19	12	18	16	4	24	11	16	17	7	22	25	21	23	2	14	25	7	15	9
	IGHV4-1	22	33	51	35	15	9	10	9	9	1	28	27	26	27	1	39	27	10	25	15	40	38	38	39	1	34	26	13	24	11
	IGHV14-1	15	28	16	20	7	3	4	5	4	1	16	10	11	12	3	19	23	16	19	4	16	20	19	18	2	20	22	19	20	2
	IGHV7-1	61	67	54	61	7	27	16	13	19	7	34	42	53	43	10	100	53	33	62	34	52	64	45	54	10	55	68	37	53	16
	IGHV2-9	68	65	97	77	18	19	34	26	26	8	37	69	72	59	19	95	55	59	70	22	67	108	91	89	21	55	95	45	65	26
	IGHV5-17	53	38	43	45	8	10	13	9	11	2	28	29	32	30	2	57	40	27	41	15	40	46	47	44	4	36	70	37	48	19
	IGHV5-15	18	13	7	13	6	8	4	3	5	3	6	4	7	6	2	17	26	15	19	6	12	21	18	17	5	25	25	22	24	2
	IGHV2-7P (VHQ52.a2!	6	4	6	5	1	0	0	0	0	0	5	5	13	8	5	4	2	4	3	1	10	2	6	6	4	7	5	2	5	3
	IGHV2-6-7	63	62	63	63	1	22	14	14	17	5	29	42	37	36	7	65	50	43	53	11	64	59	59	61	3	50	44	49	48	3
	IGHV2-6-6	1	4	5	3	2	1	1	1	1	0	1	1	2	1	1	7	0	1	3	4	0	1	1	1	1	1	0	0	0	1
	IGHV2-6-5	13	7	8	9	3	0	4	1	2	2	2	7	7	5	3	5	4	8	6	2	16	11	7	11	5	3	7	8	6	3
	IGHV2-2-2	9	9	22	13	8	1	5	2	3	2	5	11	7	8	3	24	11	11	15	8	8	14	14	12	3	21	17	7	15	7
	IGHV2-6-4	17	25	28	23	6	1	7	9	6	4	10	6	17	11	6	26	10	3	13	12	12	12	8	11	2	8	14	10	11	3
	IGHV5-6-5	26	28	31	28	3	7	3	5	5	2	17	11	10	13	4	13	19	13	15	3	19	23	30	24	6	14	23	13	17	6
	IGHV5-12-2	20	24	16	20	4	11	3	9	8	4	15	14	15	15	1	51	37	16	35	18	14	20	14	16	3	26	32	21	26	6
	IGHV5-6-4	21	14	18	18	4	7	0	7	5	4	10	14	8	11	3	41	14	29	28	14	21	22	24	22	2	36	26	15	26	11
	IGHV2-6-3	9	6	10	8	2	0	3	0	1	2	4	5	0	3	3	2	3	9	5	4	3	7	15	8	6	7	15	8	10	4
	IGHV5-6-3	6	17	26	16	10	6	6	5	6	1	4	7	2	4	3	19	9	4	11	8	11	18	7	12	6	8	11	5	8	3
	IGHV5-12-1	16	16	22	18	3	2	3	3	3	1	8	4	8	7	2	8	16	7	10	5	10	12	22	15	6	11	9	11	10	1
	IGHV5-9-5	1	8	3	4	4	1	0	1	1	1	1	0	4	2	2	7	2	0	3	4	3	2	1	2	1	1	5	4	3	2
	IGHV2-6-2	9	2	4	5	4	0	0	0	0	0	1	0	6	2	3	1	1	2	1	1	6	2	2	3	2	4	3	3	3	1
**7183/Q52**	IGHV5-6-2	16	19	25	20	5	3	3	4	3	1	7	13	15	12	4	6	17	22	15	8	11	11	15	12	2	17	27	24	23	5
	IGHV2-9-2	12	15	20	16	4	1	4	1	2	2	11	5	5	7	3	20	10	10	13	6	7	14	8	10	4	12	15	12	13	2
	IGHV5-9-4	24	24	34	27	6	8	9	4	7	3	9	5	10	8	3	25	29	26	27	2	13	13	20	15	4	29	16	21	22	7
	IGHV2-4-1	10	12	5	9	4	3	1	0	1	2	0	11	3	5	6	6	4	6	5	1	6	7	10	8	2	11	8	6	8	3
	IGHV5-9-3	32	34	23	30	6	4	15	7	9	6	6	10	10	9	2	42	29	12	28	15	23	23	17	21	3	33	41	26	33	8
	IGHV2-2-1P	20	11	12	14	5	3	0	0	1	2	1	7	8	5	4	14	15	3	11	7	10	8	15	11	4	3	19	15	12	8
	IGHV5-9-2	2	6	6	5	2	7	0	0	2	4	8	4	6	6	2	9	11	5	8	3	2	4	8	5	3	4	10	7	7	3
	IGHV2-6-1	9	12	12	11	2	2	1	0	1	1	3	6	4	4	2	7	4	3	5	2	5	6	9	7	2	5	6	2	4	2
	IGHV5-9-1	35	41	52	43	9	11	11	12	11	1	28	38	16	27	11	57	84	41	61	22	39	63	45	49	12	54	72	47	58	13
	IGHV2-6	11	7	9	9	2	1	2	2	2	1	2	8	0	3	4	5	6	12	8	4	12	11	7	10	3	4	12	4	7	5
	IGHV5-12	44	71	51	55	14	8	13	20	14	6	19	14	15	16	3	94	104	111	103	9	28	47	35	37	10	65	82	58	68	12
	IGHV5-9	4	13	7	8	5	1	2	1	1	1	6	11	5	7	3	29	16	21	22	7	6	3	7	5	2	10	15	8	11	4
	IGHV2-4	5	7	8	7	2	4	7	1	4	3	9	11	9	10	1	3	10	12	8	5	4	9	1	5	4	3	10	5	6	4
	**IGHV5-6**	**68**	**59**	**70**	**66**	**6**	**17**	**13**	**16**	**15**	**2**	**25**	**13**	**14**	**17**	**7**	**117**	**137**	**130**	**128**	**10**	**19**	**33**	**29**	**27**	**7**	**116**	**84**	**25**	**75**	**46**
	**IGHV2-3**	**81**	**104**	**87**	**91**	**12**	**32**	**46**	**40**	**39**	**7**	**41**	**44**	**32**	**39**	**6**	**164**	**173**	**148**	**162**	**13**	**33**	**31**	**46**	**37**	**8**	**117**	**112**	**54**	**94**	**35**
	**IGHV5-4**	**96**	**175**	**110**	**127**	**42**	**116**	**110**	**120**	**115**	**5**	**107**	**150**	**142**	**133**	**23**	**282**	**297**	**254**	**278**	**22**	**67**	**74**	**80**	**74**	**7**	**210**	**185**	**97**	**164**	**59**
	**IGHV2-2**	**189**	**256**	**226**	**224**	**34**	**619**	**633**	**585**	**612**	**25**	**632**	**721**	**701**	**685**	**47**	**570**	**571**	**479**	**540**	**53**	**338**	**366**	**386**	**363**	**24**	**368**	**340**	**207**	**305**	**86**
	**IGHV5-2 (VH81X)**	**322**	**400**	**403**	**375**	**46**	**5999**	**5293**	**5254**	**5515**	**419**	**3604**	**5012**	**4571**	**4396**	**720**	**1037**	**1085**	**896**	**1006**	**98**	**1559**	**1795**	**1795**	**1716**	**136**	**651**	**565**	**242**	**486**	**216**
	**IGHV5-1P**	**10**	**23**	**16**	**16**	**7**	**600**	**586**	**527**	**571**	**39**	**185**	**239**	**234**	**219**	**30**	**67**	**45**	**63**	**58**	**12**	**76**	**85**	**83**	**81**	**5**	**31**	**34**	**2**	**22**	**18**
	D usage in DJH joins																														
	**IGHD1-1 (DFL16.1)**	**1167**	**1483**	**1789**	**1480**	**311**	**22**	**26**	**29**	**26**	**4**	**513**	**612**	**579**	**568**	**50**	**534**	**790**	**613**	**646**	**131**	**826**	**985**	**892**	**901**	**80**	**584**	**533**	**301**	**473**	**151**
	**IGHD2-3**	**533**	**667**	**803**	**668**	**135**	**86**	**75**	**83**	**81**	**6**	**191**	**222**	**218**	**210**	**17**	**447**	**560**	**457**	**488**	**63**	**437**	**455**	**446**	**446**	**9**	**573**	**417**	**275**	**422**	**149**
	**IGHD2-2**	**274**	**342**	**440**	**352**	**83**	**92**	**85**	**104**	**94**	**10**	**126**	**151**	**183**	**153**	**29**	**268**	**297**	**257**	**274**	**21**	**241**	**347**	**358**	**315**	**65**	**283**	**235**	**153**	**224**	**66**
	**IGHD3-3**	**1**	**1**	**1**	**1**	**0**	**1**	**0**	**0**	**0**	**1**	**0**	**0**	**1**	**0**	**1**	**0**	**0**	**2**	**1**	**1**	**0**	**1**	**4**	**2**	**2**	**0**	**0**	**2**	**1**	**1**
	**IGHD1-2**	**329**	**475**	**580**	**461**	**126**	**69**	**42**	**69**	**60**	**16**	**181**	**190**	**147**	**173**	**23**	**314**	**467**	**351**	**377**	**80**	**413**	**487**	**347**	**416**	**70**	**560**	**419**	**268**	**416**	**146**
	**IGHD2-1**	**492**	**587**	**692**	**590**	**100**	**179**	**179**	**183**	**180**	**2**	**276**	**275**	**313**	**288**	**22**	**514**	**677**	**541**	**577**	**87**	**540**	**538**	**448**	**509**	**53**	**664**	**529**	**335**	**509**	**165**
	**IGHD2-4/2-9**	**781**	**797**	**1002**	**860**	**123**	**113**	**128**	**126**	**122**	**8**	**237**	**277**	**262**	**259**	**20**	**612**	**767**	**766**	**715**	**89**	**613**	**670**	**689**	**657**	**40**	**748**	**656**	**423**	**609**	**168**
	**IGHD2-14**	**595**	**586**	**763**	**648**	**100**	**65**	**59**	**82**	**69**	**12**	**157**	**211**	**167**	**178**	**29**	**424**	**604**	**518**	**515**	**90**	**420**	**551**	**542**	**504**	**73**	**577**	**409**	**345**	**444**	**120**
	**IGHD2-10**	**581**	**493**	**672**	**582**	**90**	**106**	**91**	**148**	**115**	**30**	**226**	**250**	**245**	**240**	**13**	**392**	**608**	**571**	**524**	**116**	**448**	**471**	**505**	**475**	**29**	**547**	**385**	**336**	**423**	**110**
	**IGHD2-11**	**396**	**414**	**464**	**425**	**35**	**99**	**104**	**97**	**100**	**4**	**130**	**145**	**151**	**142**	**11**	**328**	**365**	**366**	**353**	**22**	**295**	**331**	**316**	**314**	**18**	**375**	**307**	**276**	**319**	**51**
	**IGHD3-2**	**108**	**115**	**180**	**134**	**40**	**23**	**22**	**33**	**26**	**6**	**69**	**67**	**59**	**65**	**5**	**190**	**256**	**189**	**212**	**38**	**120**	**133**	**117**	**123**	**9**	**275**	**223**	**185**	**228**	**45**
	**IGHD4-1 (DQ52)**	**323**	**345**	**444**	**371**	**64**	**157**	**178**	**238**	**191**	**42**	**207**	**244**	**249**	**233**	**23**	**365**	**372**	**414**	**384**	**27**	**320**	**359**	**316**	**332**	**24**	**404**	**400**	**303**	**369**	**57**
	D usage in VHDJH joins																														
	**IGHD1-1 (DFL16.1)**	**500**	**561**	**576**	**546**	**40**	**237**	**220**	**183**	**213**	**28**	**669**	**893**	**825**	**795**	**115**	**413**	**309**	**315**	**346**	**59**	**488**	**524**	**574**	**528**	**43**	**300**	**329**	**211**	**280**	**62**
	**IGHD2-3**	**265**	**286**	**275**	**276**	**11**	**501**	**448**	**405**	**452**	**48**	**491**	**596**	**573**	**553**	**56**	**388**	**337**	**237**	**320**	**77**	**372**	**355**	**394**	**374**	**20**	**275**	**403**	**181**	**286**	**112**
	**IGHD2-2**	**149**	**118**	**116**	**128**	**19**	**303**	**318**	**228**	**283**	**48**	**259**	**281**	**279**	**273**	**12**	**248**	**152**	**118**	**173**	**67**	**164**	**191**	**173**	**176**	**14**	**163**	**207**	**87**	**152**	**61**
	**IGHD3-3**	**79**	**79**	**64**	**74**	**8**	**193**	**218**	**215**	**209**	**14**	**121**	**222**	**236**	**193**	**63**	**149**	**124**	**116**	**129**	**17**	**101**	**120**	**120**	**114**	**11**	**106**	**95**	**35**	**79**	**38**
	**IGHD1-2**	**236**	**284**	**260**	**260**	**24**	**491**	**464**	**448**	**468**	**22**	**346**	**460**	**410**	**405**	**58**	**392**	**298**	**237**	**309**	**78**	**286**	**359**	**362**	**336**	**43**	**286**	**447**	**190**	**308**	**130**
	**IGHD2-1**	**157**	**137**	**149**	**148**	**10**	**544**	**389**	**420**	**451**	**82**	**313**	**366**	**288**	**323**	**40**	**216**	**185**	**199**	**200**	**15**	**170**	**221**	**287**	**226**	**59**	**174**	**206**	**160**	**180**	**24**
	**IGHD2-4/2-9**	**404**	**446**	**433**	**428**	**22**	**1078**	**935**	**1044**	**1019**	**75**	**720**	**954**	**891**	**855**	**121**	**814**	**625**	**492**	**644**	**162**	**518**	**646**	**511**	**558**	**76**	**534**	**595**	**328**	**485**	**140**
	**IGHD2-14**	**432**	**580**	**544**	**519**	**77**	**1317**	**1232**	**1161**	**1237**	**78**	**929**	**1105**	**1106**	**1046**	**102**	**823**	**693**	**542**	**686**	**140**	**602**	**740**	**763**	**702**	**87**	**730**	**677**	**400**	**602**	**178**
	**IGHD2-10**	**292**	**362**	**277**	**310**	**45**	**1185**	**1176**	**1041**	**1134**	**80**	**735**	**946**	**787**	**823**	**110**	**557**	**499**	**433**	**496**	**62**	**395**	**508**	**537**	**480**	**75**	**382**	**427**	**233**	**347**	**102**
	**IGHD2-11**	**175**	**158**	**165**	**166**	**9**	**617**	**587**	**576**	**594**	**21**	**317**	**570**	**486**	**458**	**129**	**390**	**356**	**242**	**329**	**78**	**306**	**300**	**207**	**271**	**55**	**242**	**200**	**136**	**193**	**53**
	**IGHD3-2**	**110**	**115**	**145**	**123**	**19**	**353**	**321**	**378**	**351**	**29**	**262**	**297**	**337**	**299**	**37**	**216**	**158**	**140**	**171**	**40**	**138**	**178**	**186**	**167**	**26**	**122**	**220**	**89**	**144**	**68**
	**IGHD4-1 (DQ52)**	**172**	**172**	**169**	**171**	**2**	**621**	**546**	**541**	**570**	**45**	**360**	**410**	**347**	**372**	**33**	**369**	**267**	**219**	**285**	**77**	**237**	**261**	**227**	**241**	**18**	**193**	**300**	**192**	**228**	**62**
	D usage in DJH+VHDJH joins																														
	**IGHD1-1 (DFL16.1)**	**1667**	**2044**	**2365**	**2025**	**349**	**259**	**246**	**212**	**239**	**24**	**1182**	**1505**	**1404**	**1363**	**165**	**947**	**1099**	**928**	**991**	**94**	**1314**	**1509**	**1466**	**1429**	**103**	**884**	**862**	**512**	**753**	**209**
	**IGHD2-3**	**798**	**953**	**1078**	**943**	**140**	**587**	**523**	**488**	**533**	**50**	**682**	**818**	**791**	**764**	**72**	**835**	**897**	**694**	**808**	**104**	**809**	**810**	**840**	**820**	**18**	**848**	**820**	**456**	**708**	**219**
	**IGHD2-2**	**423**	**460**	**556**	**480**	**69**	**395**	**403**	**332**	**377**	**39**	**385**	**432**	**462**	**426**	**39**	**516**	**449**	**375**	**447**	**70**	**405**	**538**	**531**	**491**	**75**	**446**	**442**	**240**	**376**	**118**
	**IGHD3-3**	**80**	**80**	**65**	**75**	**8**	**194**	**218**	**215**	**209**	**13**	**121**	**222**	**237**	**193**	**63**	**149**	**124**	**118**	**130**	**16**	**101**	**121**	**124**	**115**	**12**	**106**	**95**	**37**	**79**	**37**
	**IGHD1-2**	**565**	**759**	**840**	**721**	**142**	**560**	**506**	**517**	**528**	**28**	**527**	**650**	**557**	**578**	**65**	**706**	**765**	**588**	**686**	**90**	**699**	**846**	**709**	**751**	**82**	**846**	**866**	**458**	**723**	**230**
	**IGHD2-1**	**649**	**724**	**841**	**738**	**97**	**723**	**568**	**603**	**632**	**81**	**589**	**641**	**601**	**611**	**27**	**730**	**862**	**740**	**777**	**74**	**710**	**759**	**735**	**735**	**25**	**838**	**735**	**495**	**689**	**176**
	**IGHD2-4/2-9**	**1185**	**1243**	**1435**	**1288**	**131**	**1191**	**1063**	**1170**	**1141**	**69**	**957**	**1231**	**1153**	**1113**	**141**	**1426**	**1392**	**1258**	**1359**	**89**	**1131**	**1316**	**1200**	**1216**	**94**	**1282**	**1251**	**751**	**1094**	**298**
	**IGHD2-14**	**1027**	**1166**	**1307**	**1167**	**140**	**1382**	**1291**	**1243**	**1306**	**70**	**1086**	**1316**	**1273**	**1225**	**123**	**1247**	**1297**	**1060**	**1202**	**125**	**1022**	**1291**	**1305**	**1206**	**159**	**1307**	**1086**	**745**	**1046**	**283**
	**IGHD2-10**	**873**	**855**	**949**	**892**	**50**	**1291**	**1267**	**1189**	**1249**	**53**	**961**	**1196**	**1032**	**1063**	**121**	**949**	**1107**	**1004**	**1020**	**80**	**843**	**979**	**1042**	**955**	**102**	**929**	**812**	**569**	**770**	**184**
	**IGHD2-11**	**571**	**572**	**629**	**591**	**33**	**716**	**691**	**673**	**694**	**22**	**447**	**715**	**637**	**600**	**138**	**718**	**721**	**608**	**682**	**64**	**601**	**631**	**523**	**585**	**56**	**617**	**507**	**412**	**512**	**103**
	**IGHD3-2**	**218**	**230**	**325**	**258**	**59**	**376**	**343**	**411**	**377**	**34**	**331**	**364**	**396**	**364**	**32**	**406**	**414**	**329**	**383**	**47**	**258**	**311**	**303**	**291**	**29**	**397**	**443**	**274**	**371**	**87**
	**IGHD4-1 (DQ52)**	**495**	**517**	**613**	**542**	**63**	**778**	**724**	**779**	**761**	**31**	**567**	**654**	**596**	**606**	**45**	**734**	**639**	**633**	**669**	**57**	**557**	**620**	**543**	**573**	**41**	**597**	**700**	**495**	**597**	**103**

Notes:
1. Data were nomalized to 78,091 total reads.
2. Proximal V and D segments are denoted in bold.

Supplementary Table 2 | Productive and non-productive VHDJH junctions in WT and IGCR1/CBEs mutated pro-B cells.																																					
		WT (n=3)						CBE1&2 -/- (n=3)						CBE1 -/- (n=3)						CBE2 -/- (n=3)						CBE1 inv/inv (n=3)						CBE2 inv/inv (n=3)					
		Productive			Non-productive			Productive			Non-productive																										
**VH domains**		#1	#2	#3	#1	#2	#3	#1	#2	#3	#1	#2	#3	#1	#2	#3	#1	#2	#3	#1	#2	#3	#1	#2	#3	#1	#2	#3	#1	#2	#3	#1	#2	#3	#1	#2	#3
	Ighv1-84	11	14	10	13	16	15	1	0	7	1	0	6	4	3	2	1	5	5	16	2	3	6	3	2	3	2	6	8	13	7	6	12	6	6	8	0
	Ighv1-82	1	1	2	1	3	1	1	1	0	0	2	1	1	1	0	2	2	0	5	0	2	1	0	0	2	2	0	3	1	0	4	3	0	1	1	2
	Ighv1-81	0	3	6	3	1	2	2	0	1	1	0	0	0	3	0	1	0	2	2	0	0	1	0	0	3	2	2	2	4	1	4	3	3	2	2	2
	Ighv1-80	12	10	6	6	12	13	2	1	1	0	3	1	4	3	5	5	2	0	12	4	4	22	1	0	10	8	12	6	4	3	11	22	12	7	11	7
	Ighv1-78	7	3	2	3	3	3	1	0	1	0	0	0	2	4	2	0	2	0	1	1	2	0	1	0	5	2	6	2	3	4	7	7	9	6	7	1
	Ighv1-77	9	4	4	7	4	7	1	1	2	1	2	1	3	3	3	0	3	2	10	2	2	1	3	0	0	4	10	5	4	9	14	10	5	5	6	2
	Ighv1-76	7	14	1	3	7	10	2	0	1	2	1	0	9	3	1	0	5	2	16	13	2	8	7	2	7	4	5	4	3	11	17	22	9	6	5	6
	Ighv1-75	6	0	3	3	1	0	0	0	2	1	0	0	2	2	0	1	1	0	3	3	0	1	0	0	1	1	2	2	3	2	0	5	2	3	1	0
	Ighv1-74	3	3	1	0	1	2	0	0	0	0	0	0	3	2	0	1	0	4	2	2	0	1	1	0	0	3	2	2	4	4	0	5	1	1	5	4
	Ighv1-72	1	1	0	0	0	0	0	0	0	0	0	0	0	0	1	0	0	0	0	0	0	0	0	0	0	0	0	1	1	0	0	1	0	0	1	0
	Ighv1-71	1	1	0	0	0	0	0	0	0	0	0	1	1	0	1	0	0	0	0	0	0	0	0	0	0	3	1	0	0	0	0	0	0	0	3	0
	Ighv8-12	28	18	31	14	22	22	9	6	15	5	6	6	15	18	18	5	8	4	49	21	11	16	14	11	16	22	19	9	8	13	34	44	29	15	22	19
	Ighv1-69	2	7	4	4	4	4	1	0	0	0	0	0	3	3	1	1	2	3	2	3	2	0	1	0	3	9	6	3	9	3	0	3	1	3	6	0
	Ighv1-67	30	31	20	28	26	38	8	10	8	2	5	1	17	11	8	7	15	8	41	18	27	23	14	14	22	17	15	19	8	21	17	54	37	21	27	15
**J558/3609**	Ighv1-66	1	5	0	6	1	1	0	0	0	0	0	0	5	0	2	0	0	0	0	3	0	3	1	0	2	2	1	0	0	1	1	1	0	1	1	3
	Ighv8-11	13	13	14	6	10	20	4	1	3	3	0	2	7	7	7	5	5	4	57	15	6	10	4	8	10	12	5	3	9	9	13	17	29	7	14	7
	Ighv1-64	17	26	10	11	13	20	8	3	4	0	2	1	10	12	14	5	7	7	52	13	9	23	7	14	17	23	21	18	15	19	23	37	17	23	37	5
	Ighv1-63	3	10	6	1	10	3	1	4	1	1	0	1	2	3	1	0	1	2	11	7	2	5	3	3	3	4	0	1	3	3	3	14	2	4	9	6
	Ighv1-62-3	4	5	7	3	4	3	1	1	0	1	2	4	3	2	4	4	2	0	6	0	3	4	1	1	3	4	0	3	2	4	8	5	5	2	3	2
	Ighv1-61	6	4	3	4	3	0	0	0	0	0	1	0	3	4	1	2	0	0	3	0	1	4	2	1	3	5	3	0	1	5	1	6	4	2	4	0
	Ighv1-59	4	6	3	5	2	4	0	1	4	0	1	0	4	1	2	1	0	0	4	5	2	1	0	0	1	6	4	1	1	6	3	6	3	3	3	1
	Ighv1-58	2	0	1	3	0	1	0	0	0	0	0	0	0	1	1	0	0	1	0	0	0	0	0	0	2	0	0	0	0	1	2	2	2	0	0	3
	Ighv8-8	0	2	2	2	1	0	0	0	0	1	0	0	2	1	1	0	0	1	0	0	0	0	0	0	0	1	1	0	0	1	1	1	0	0	0	1
	Ighv1-56	1	1	0	3	8	4	0	0	0	0	0	0	2	0	0	0	1	3	1	2	0	6	0	2	2	2	1	0	1	0	1	0	4	1	3	0
	Ighv1-55	16	9	10	10	11	10	1	5	4	1	2	1	5	5	5	5	6	1	24	9	3	16	4	3	6	14	6	9	5	9	8	19	16	13	18	5
	Ighv1-54	15	20	22	20	29	15	2	7	2	1	2	5	11	20	17	6	3	6	15	14	15	27	4	7	23	21	15	11	12	13	25	34	32	21	15	2
	Ighv1-53	7	5	15	4	8	4	0	3	3	1	2	1	6	5	2	0	3	0	3	2	2	2	0	0	8	9	4	5	5	2	3	2	0	6	0	2
	Ighv1-52	3	2	3	1	6	1	0	1	0	0	0	0	4	3	1	1	0	2	5	5	0	0	2	4	2	2	0	2	3	3	2	7	7	4	1	2
	Ighv1-50	2	2	2	1	1	0	0	0	0	0	1	0	0	1	0	0	2	1	2	1	2	3	1	0	0	1	1	0	1	6	4	2	2	1	2	1
	Ighv8-4	1	2	1	6	4	7	0	0	0	0	1	1	1	1	1	0	1	3	2	3	3	1	5	2	0	3	2	2	4	3	2	0	0	3	0	2
	Ighv1-47	13	4	6	31	14	13	1	1	0	1	0	1	1	1	4	2	6	7	4	5	3	17	11	16	2	3	8	9	8	19	9	2	1	18	10	8
	Ighv1-43	1	7	2	7	4	3	2	0	0	0	0	0	3	2	0	2	2	2	5	5	0	4	2	2	3	3	1	3	4	4	0	5	0	2	7	4
	Ighv1-42	13	10	6	9	9	13	4	2	5	1	2	4	5	6	6	2	9	3	18	16	10	11	9	6	6	12	15	9	12	14	8	19	12	12	16	6
	Ighv1-39	3	11	5	10	2	6	4	8	2	0	0	2	7	3	6	1	5	0	10	12	2	7	6	5	8	4	7	5	6	3	10	14	5	12	6	1
	Ighv1-37	3	0	1	2	2	2	1	1	1	0	0	0	2	4	2	0	0	0	3	1	1	1	0	1	2	1	2	2	4	0	2	3	1	2	1	0
	Ighv1-34	5	5	10	8	5	11	0	4	0	0	0	0	3	3	1	4	4	3	10	2	3	13	1	2	7	5	7	6	15	6	4	15	2	5	5	0
	Ighv1-26	21	14	14	13	13	11	2	3	1	3	0	2	4	6	6	4	4	6	29	9	12	16	12	2	8	11	7	11	12	12	10	32	11	21	8	8
	Ighv1-23	2	4	1	0	1	0	0	1	1	0	1	0	4	1	0	0	0	0	1	6	0	1	0	0	2	4	1	6	5	0	2	3	2	0	9	1
	Ighv1-22	2	3	1	1	1	0	1	1	0	0	0	0	0	1	0	0	0	0	2	6	1	0	4	0	0	0	1	0	1	0	3	2	2	2	1	1
	Ighv1-19	6	13	6	9	2	6	1	1	0	0	0	0	8	1	1	1	1	2	14	9	6	7	2	2	5	8	6	5	7	6	7	15	12	3	7	3
**J558**	Ighv1-18	17	7	19	12	21	8	3	3	0	0	2	1	9	9	3	5	3	1	37	14	9	15	8	2	12	6	12	18	9	9	5	28	19	14	18	11
	Ighv1-14	18	25	22	27	22	22	3	9	3	2	1	8	17	11	17	6	10	17	31	12	15	15	25	25	8	17	29	15	13	25	26	60	26	27	27	23
	Ighv1-12	13	11	11	7	9	9	3	0	4	0	3	1	6	6	7	0	4	1	6	4	3	8	3	5	6	2	7	1	7	6	9	8	6	1	7	4
	Ighv1-9	37	40	34	29	29	21	12	9	8	5	7	4	7	12	19	13	12	7	48	11	11	41	6	21	22	40	39	14	23	26	31	55	30	36	22	14
	Ighv15-2	12	25	15	47	33	29	3	5	2	2	4	2	12	14	12	18	10	16	20	15	16	56	36	13	11	25	14	24	25	31	13	29	20	31	27	9
	Ighv1-7	1	1	0	0	0	0	0	0	0	0	0	0	0	0	1	0	0	0	0	0	0	0	0	0	0	0	2	0	0	0	0	0	0	0	0	0
	Ighv10-3	20	8	8	20	24	18	2	4	2	3	1	4	3	0	10	12	10	9	9	5	2	26	9	20	4	8	6	17	11	32	8	10	5	18	33	13
	Ighv1-5	30	33	33	30	17	32	3	7	16	4	1	0	21	17	12	12	16	16	65	28	23	41	9	22	18	16	23	27	21	26	30	55	38	30	29	14
	Ighv1-4	21	20	15	26	9	11	8	4	8	2	0	5	8	12	12	6	11	10	41	41	6	34	3	10	14	19	17	11	21	14	25	26	19	5	27	15
	Ighv1-2P	23	18	15	50	36	40	4	5	3	4	10	11	15	14	6	14	18	22	33	21	15	51	22	30	16	21	11	31	35	29	23	35	23	46	50	26
	Ighv8-2	12	11	9	44	41	29	1	0	2	5	4	7	2	1	4	22	17	14	16	10	3	33	22	22	8	16	4	35	37	42	10	18	4	20	47	27
	IGHV9-4	13	9	11	10	12	12	2	0	4	3	1	8	3	8	6	2	2	5	18	10	16	18	4	7	13	22	16	7	11	17	9	12	10	11	8	3
	IGHV14-4	14	15	13	10	26	26	5	5	6	3	7	2	13	10	11	5	6	8	14	21	2	10	8	8	13	20	15	21	27	18	16	39	29	18	23	16
	IGHV7-3	21	26	29	13	20	14	5	7	6	6	4	2	21	16	15	7	7	11	35	14	14	32	11	10	24	19	19	14	11	17	21	34	11	16	13	9
	IGHV9-2-1	25	38	22	22	27	15	10	5	3	3	5	5	17	16	10	6	5	10	37	12	27	27	19	13	34	34	27	22	21	16	33	50	17	22	8	15
	IGHV9-3-1	18	19	14	13	9	15	3	5	4	3	1	1	13	20	12	7	3	7	22	21	4	8	2	10	10	7	13	10	15	18	15	36	12	10	9	2
	IGHV12-1-1	11	10	7	9	10	12	0	2	1	3	3	0	2	7	3	11	8	2	9	7	6	7	7	1	6	9	3	7	7	8	6	8	5	14	8	3
	IGHV9-3	9	5	6	7	3	7	3	4	2	3	1	2	5	2	6	5	1	3	9	1	12	6	3	0	8	14	8	6	3	12	3	17	5	3	5	4
	IGHV9-2	0	4	2	11	3	10	0	0	0	0	0	0	2	0	2	0	9	0	0	3	3	6	9	8	2	3	1	10	4	7	2	2	2	10	7	3
**Middle**	IGHV9-1	6	8	7	5	8	13	2	2	2	2	0	2	2	3	4	3	1	0	13	6	3	7	5	1	6	4	5	6	11	7	12	18	7	3	3	3
	IGHV14-3	22	33	25	16	35	23	3	12	6	5	14	4	24	32	12	14	9	7	23	40	11	15	5	2	32	52	38	12	36	24	24	53	11	27	8	7
	IGHV11-2	10	7	7	26	23	22	2	0	4	4	2	11	3	5	4	9	18	22	9	16	2	19	20	16	6	8	2	20	16	13	4	9	8	21	20	16
	IGHV3-2	25	35	41	35	37	36	7	22	9	4	8	8	28	25	17	9	23	16	38	7	10	24	5	12	32	51	54	27	33	30	18	31	14	24	9	7
	IGHV4-2	7	8	11	14	11	13	6	1	1	8	2	3	1	11	3	11	10	12	4	7	2	13	7	3	3	11	5	10	18	23	3	4	2	15	15	3
	IGHV3-1	10	11	8	19	8	13	4	7	2	0	2	2	9	8	10	10	4	8	13	6	12	11	5	4	14	11	8	8	14	13	7	17	4	7	8	3
	IGHV4-1	15	14	30	7	19	21	5	6	7	4	4	2	16	15	16	12	12	10	24	15	9	15	10	1	24	17	21	16	20	17	17	18	7	17	8	6
	IGHV14-1	5	12	2	10	15	11	2	1	2	1	3	3	13	9	8	3	1	3	14	20	11	5	3	5	4	10	13	12	10	6	13	17	11	7	5	8
	IGHV7-1	37	33	25	23	34	28	23	6	8	3	10	5	20	26	31	14	16	22	67	31	17	33	22	16	28	35	18	24	29	26	43	47	30	12	21	7
	IGHV2-9	30	29	56	38	36	40	12	19	21	7	15	4	27	31	35	10	38	37	64	31	30	31	24	29	38	57	48	29	50	43	28	64	25	26	31	20
	IGHV5-17	29	15	13	24	23	30	5	3	7	5	10	2	23	18	15	5	10	16	31	23	19	25	17	8	18	25	27	22	21	20	22	37	26	14	33	11
	IGHV5-15	6	4	1	12	9	6	5	3	0	3	1	3	5	4	3	1	0	4	12	14	1	5	12	14	4	17	7	8	4	11	9	17	13	16	8	9
	IGHV2-6-7	30	41	29	33	21	34	14	10	10	8	4	4	22	19	23	7	23	14	39	38	25	26	9	18	32	31	29	32	27	30	28	33	33	22	11	16
	IGHV2-6-6	0	4	4	0	0	1	0	0	0	1	1	1	1	0	2	0	1	0	5	0	1	2	0	0	0	1	1	0	0	0	1	0	0	0	0	0
	IGHV2-6-5	9	1	6	4	6	2	0	4	0	0	0	1	1	5	4	1	2	3	3	3	7	2	1	1	14	7	3	2	4	4	3	5	3	0	2	5
	IGHV2-2-2	4	4	5	5	5	17	0	5	1	1	0	1	4	6	5	1	5	2	15	8	11	9	3	0	6	5	9	2	8	5	14	12	6	7	5	1
	IGHV2-6-4	10	10	13	7	15	15	0	2	5	1	3	4	8	2	11	2	4	6	17	9	2	9	1	1	7	7	5	5	5	3	5	7	8	3	7	2
	IGHV5-6-5	11	10	14	15	18	17	5	3	5	2	0	0	11	8	8	6	3	2	11	14	13	2	5	0	11	11	17	8	12	13	7	14	4	7	9	9
	IGHV5-12-2	13	16	6	7	8	10	6	3	7	5	0	2	12	12	6	3	2	9	38	22	13	13	15	3	9	11	9	5	9	5	17	14	17	9	18	4
	IGHV5-6-4	9	11	12	11	3	6	2	0	2	5	0	5	6	9	7	4	5	1	33	8	23	8	6	6	12	15	13	9	7	11	13	19	11	23	7	4
	IGHV2-6-3	1	2	2	8	4	8	0	1	0	0	2	0	1	1	0	3	4	0	0	0	0	2	3	9	1	0	3	2	7	10	2	1	0	5	14	8
	IGHV5-6-3	3	12	9	3	5	17	1	2	2	3	4	3	3	1	0	1	6	2	16	4	2	3	5	2	2	4	4	9	14	3	3	5	4	5	6	1
	IGHV5-12-1	7	7	12	9	9	10	1	0	2	1	3	1	6	2	6	2	2	2	6	8	4	2	7	3	4	6	15	6	6	7	2	7	5	9	2	6
	IGHV5-9-5	0	1	1	1	7	2	0	0	1	1	0	0	1	0	2	0	0	2	7	2	0	0	0	0	3	1	1	0	1	0	0	3	0	1	2	4
	IGHV2-6-2	7	0	1	2	2	3	0	0	0	0	0	0	1	0	1	0	0	5	1	0	1	0	1	1	1	0	1	5	2	1	3	0	3	1	3	0
**7183/Q52**	IGHV5-6-2	7	11	14	9	8	11	2	2	2	1	1	2	7	9	6	0	4	9	2	5	7	4	12	15	5	7	10	6	4	5	9	18	14	8	9	10
	IGHV2-9-2	2	7	15	10	5	5	1	2	1	0	2	0	6	4	4	5	1	1	2	5	4	18	5	4	6	10	5	1	4	3	6	13	6	6	2	6
	IGHV5-9-4	8	8	18	16	16	16	2	8	0	6	1	4	7	3	7	2	2	2	22	15	12	3	14	14	4	9	6	8	4	14	13	9	7	16	7	14
	IGHV2-4-1	6	5	1	4	7	4	0	1	0	3	0	0	0	6	0	0	5	3	5	1	3	1	3	0	2	1	5	4	6	5	5	4	3	6	4	3
	IGHV5-9-3	22	15	8	10	19	15	3	11	6	1	4	1	3	5	5	3	5	5	14	20	4	28	9	8	11	15	10	12	8	7	21	29	14	12	12	12
	IGHV5-9-2	2	4	4	0	2	2	5	0	0	2	0	0	5	4	5	3	0	1	6	9	4	3	2	1	1	0	5	1	4	3	2	7	2	2	3	5
	IGHV2-6-1	3	4	5	5	8	7	0	0	0	2	1	0	2	3	1	0	3	3	1	3	1	6	1	2	1	3	4	4	2	4	0	1	2	4	5	0
	IGHV5-9-1	13	19	21	22	22	31	9	6	8	2	5	4	21	23	6	7	15	10	22	47	15	35	37	26	17	40	28	22	23	17	32	46	32	22	26	15
	IGHV2-6	2	4	1	7	3	7	0	1	0	1	1	2	2	5	0	0	3	0	0	2	3	5	4	9	4	2	2	8	9	5	1	3	2	3	9	2
	IGHV5-12	23	28	24	21	43	27	5	9	13	3	4	7	10	5	10	9	9	5	38	66	61	56	38	49	7	19	12	21	26	22	19	44	32	46	38	26
	IGHV5-9	2	4	7	2	9	0	0	0	0	1	2	1	5	6	3	1	5	2	9	14	1	20	2	20	4	1	6	2	2	1	2	8	4	8	7	4
	IGHV2-4	5	2	3	0	5	5	2	2	1	2	5	0	3	3	1	6	8	6	1	1	4	2	8	8	0	2	0	3	7	1	0	7	1	3	3	4
	IGHV5-6	11	14	11	57	45	59	7	7	7	10	5	9	16	4	5	9	9	9	23	52	49	94	84	81	2	15	17	17	18	12	24	30	9	92	54	16
	IGHV2-3	27	38	16	52	65	71	8	13	14	24	33	26	12	16	8	29	27	24	42	55	43	120	115	102	14	14	17	19	17	27	23	44	29	94	67	25
	IGHV5-4	30	38	25	65	136	85	21	18	24	95	90	92	30	28	30	73	121	109	58	117	100	221	178	153	15	12	24	52	62	56	38	55	39	170	129	58
	IGHV2-2	43	44	41	144	211	182	57	89	73	559	539	509	109	103	71	520	611	627	132	163	110	434	405	369	60	58	83	277	303	297	89	78	66	276	259	141
	IGHV5-2	47	66	71	273	335	328	940	879	896	5019	4380	4327	655	874	751	2932	4110	3798	175	224	136	862	859	758	233	283	305	1312	1502	1478	129	93	43	522	468	199

Notes:
1. Data were nomalized to 78,091 total reads.
2. Pseudo V are not listed in this table.

Supplementary Table 3 | Absolute utilization of Igh VH and D segments in single Igh WAPL-degron v-Abl pro-B cells.

		WT untreated (n=3)					WT+Dox/IAA (n=3)					3'CBE del untreated (n=3)					3'CBE del+Dox/IAA (n=3)					3'CBE inv untreated (n=3)					3'CBE inv +Dox/IAA (n=3)				
**VH domains**		#1	#2	#3	Average	s.d.	#1	#2	#3	Average	s.d.	#1	#2	#3	Average	s.d.	#1	#2	#3	Average	s.d.	#1	#2	#3	Average	s.d.	#1	#2	#3	Average	s.d.
	Ighv1-86P	0	0	0	0	0	0	0	5	2	3	0	0	0	0	0	0	2	2	1	1	0	0	0	0	0	0	3	0	1	2
	Ighv1-85	1	0	0	0	1	17	18	19	18	1	0	0	0	0	0	7	6	2	5	3	0	0	4	1	2	6	4	1	4	3
	Ighv1-84	0	0	0	0	0	0	0	8	3	5	0	0	0	0	0	2	1	4	2	2	0	0	0	0	0	0	1	0	0	1
	Ighv1-83P	0	0	0	0	0	5	2	0	2	3	0	0	0	0	0	1	4	3	3	2	0	0	1	0	1	0	2	4	2	2
	Ighv1-82	0	0	0	0	0	4	9	21	11	9	0	0	0	0	0	8	7	5	7	2	0	0	0	0	0	4	10	9	8	3
	Ighv1-81	0	0	0	0	0	1	12	8	7	6	0	0	0	0	0	11	8	5	8	3	0	4	0	1	2	6	5	7	6	1
	Ighv1-80	0	0	1	0	1	8	7	14	10	4	0	0	0	0	0	10	6	1	6	5	0	0	1	0	1	1	11	0	4	6
	Ighv1-79P	0	0	0	0	0	1	8	2	4	4	1	0	0	0	1	0	1	2	1	1	0	0	0	0	0	0	5	0	2	3
	Ighv1-78	0	0	8	3	5	28	50	48	42	12	1	8	0	3	4	23	18	26	22	4	12	8	9	10	2	14	49	29	31	18
	Ighv1-77	0	0	0	0	0	4	3	22	10	11	0	0	1	0	1	3	7	3	4	2	0	0	0	0	0	2	3	5	3	2
	Ighv1-76	0	0	5	2	3	42	35	23	33	10	1	0	3	1	2	16	19	28	21	6	0	1	4	2	2	27	25	45	32	11
	Ighv1-75	0	0	0	0	0	14	18	22	18	4	0	0	0	0	0	2	1	17	7	9	0	0	0	0	0	8	7	23	13	9
	Ighv1-74	0	1	4	2	2	36	51	38	42	8	4	4	4	4	0	12	13	14	13	1	5	5	6	5	1	20	24	33	26	7
	Ighv1-73P	0	3	0	1	2	16	18	36	23	11	0	0	0	0	0	4	7	9	7	3	0	0	0	0	0	2	6	10	6	4
	Ighv1-72	0	0	1	0	1	4	7	13	8	5	0	0	1	0	1	4	7	2	4	3	1	7	0	3	4	3	24	5	11	12
	Ighv1-71	0	0	0	0	0	0	0	0	0	0	0	0	0	0	0	0	0	0	0	0	0	0	0	0	0	0	0	0	0	0
	Ighv1-70P	2	0	4	2	2	95	73	102	90	15	1	1	10	4	5	44	22	40	35	12	0	4	5	3	3	45	56	86	62	21
	Ighv8-12	19	3	12	11	8	91	101	79	90	11	8	2	4	5	3	28	27	40	32	7	1	19	11	10	9	61	80	86	76	13
	Ighv1-69	0	0	2	1	1	6	13	13	11	4	5	0	1	2	3	8	9	6	8	2	1	5	2	3	2	8	17	8	11	5
	Ighv1-67	2	0	0	1	1	13	13	13	13	0	0	0	3	1	2	8	1	1	3	4	0	1	3	1	2	12	4	10	9	4
	Ighv1-66	0	0	0	0	0	1	1	6	3	3	0	0	0	0	0	0	0	1	0	1	0	0	0	0	0	1	0	1	1	1
J558/3609	Ighv8-11	1	5	1	2	2	37	23	35	32	8	2	2	11	5	5	27	20	15	21	6	2	2	7	4	3	26	25	32	28	4
	Ighv1-64	0	0	0	0	0	9	4	12	8	4	2	2	3	2	1	8	17	2	9	8	1	0	1	1	1	7	4	7	6	2
	Ighv1-63	2	0	2	1	1	19	29	32	27	7	0	1	6	2	3	4	10	17	10	7	1	0	1	1	1	19	29	22	23	5
	Ighv8-9	0	0	0	0	0	19	8	10	12	6	0	0	0	0	0	0	7	5	4	4	2	0	3	2	2	2	3	12	6	6
	Ighv1-62-3	1	1	0	1	1	3	3	3	3	0	0	0	1	0	1	3	0	1	1	2	0	0	2	1	1	2	2	3	2	1
	Ighv1-62-2	0	0	0	0	0	0	0	0	0	0	0	0	0	0	0	0	0	0	0	0	0	0	0	0	0	1	0	0	0	1
	Ighv1-62P	0	0	2	1	1	1	6	7	5	3	0	0	0	0	0	0	3	3	2	2	0	0	1	0	1	2	1	1	1	1
	Ighv1-61	1	4	1	2	2	23	19	26	23	4	1	1	4	2	2	8	14	6	9	4	4	8	7	6	2	21	21	20	21	1
	Ighv1-59	0	0	0	0	0	9	12	11	11	2	2	0	1	1	1	3	4	5	4	1	2	3	1	2	1	3	13	8	8	5
	Ighv1-58	3	0	0	1	2	23	28	30	27	4	0	0	1	0	1	13	14	9	12	3	4	0	5	3	3	15	11	13	13	2
	Ighv8-8	2	9	2	4	4	60	73	72	68	7	11	5	8	8	3	23	30	46	33	12	29	18	20	22	6	41	45	64	50	12
	Ighv8-7P	3	0	9	4	5	29	23	29	27	3	0	4	4	3	2	32	24	29	28	4	7	3	7	6	2	27	23	37	29	7
	Ighv1-56	0	0	1	0	1	2	3	5	3	2	0	0	0	0	0	0	2	1	1	1	0	0	1	0	1	1	1	5	2	2
	Ighv1-55	0	1	2	1	1	10	7	11	9	2	0	0	0	0	0	4	0	6	3	3	0	3	3	2	2	3	2	10	5	4
	Ighv1-54	1	5	7	4	3	46	47	34	42	7	5	9	8	7	2	7	26	26	20	11	12	14	3	10	6	19	33	38	30	10
	Ighv8-6	0	0	2	1	1	21	13	5	13	8	0	0	2	1	1	6	11	1	6	5	0	4	5	3	3	8	7	9	8	1
	Ighv1-53	0	1	2	1	1	17	4	8	10	7	0	6	1	2	3	8	8	7	8	1	1	1	1	1	0	3	7	15	8	6
	Ighv1-52	1	2	4	2	2	41	13	14	23	16	2	1	1	1	1	15	16	13	15	2	6	7	3	5	2	20	13	19	17	4
	Ighv1-51P	0	0	0	0	0	2	0	0	1	1	7	0	0	2	4	0	0	1	0	1	0	0	0	0	0	3	0	0	1	2
	Ighv1-50	0	0	0	0	0	3	5	9	6	3	0	2	3	2	2	1	1	2	1	1	0	1	2	1	1	2	5	5	4	2
	Ighv8-5	0	0	0	0	0	8	3	1	4	4	0	0	0	0	0	5	0	2	2	3	0	1	8	3	4	2	0	3	2	2
	Ighv1-49	0	0	0	0	0	2	5	3	3	2	0	1	3	1	2	0	7	2	3	4	0	4	0	1	2	8	1	6	5	4
	Ighv8-4	0	0	0	0	0	15	12	16	14	2	0	0	3	1	2	3	14	6	8	6	1	1	0	1	1	14	3	8	8	6
	Ighv1-47	8	15	15	13	4	40	42	39	40	2	1	7	6	5	3	25	25	19	23	3	16	14	25	18	6	35	33	27	32	4
	Ighv1-46P	0	0	0	0	0	5	0	0	2	3	4	0	0	1	2	1	3	2	2	1	0	0	0	0	0	0	4	0	1	2
	Ighv1-43	0	0	0	0	0	3	0	0	1	2	0	0	0	0	0	1	0	0	0	1	0	2	0	1	1	3	0	7	3	4
	Ighv1-42	3	0	1	1	2	11	25	28	21	9	2	0	1	1	1	1	11	0	4	6	13	1	2	5	7	6	1	2	3	3
	Ighv1-40P	0	0	0	0	0	4	0	0	1	2	0	0	0	0	0	0	0	0	0	0	2	0	5	2	3	0	0	0	0	0
	Ighv1-39	3	0	3	2	2	21	12	24	19	6	0	2	3	2	2	7	12	6	8	3	8	1	6	5	4	7	9	23	13	9
	Ighv1-38P	0	0	2	1	1	1	2	1	1	1	0	1	1	1	1	0	0	0	0	0	0	0	0	0	0	1	0	1	1	1
	Ighv1-36	0	0	1	0	1	2	1	0	1	1	0	0	0	0	0	0	0	4	1	2	0	0	0	0	0	0	0	0	0	0
	Ighv1-34	0	0	0	0	0	0	1	0	0	1	0	0	0	0	0	4	1	0	2	2	0	0	0	0	0	0	0	0	0	0
	Ighv1-33P	0	0	0	0	0	1	0	4	2	2	0	0	1	0	1	0	1	2	1	1	0	0	0	0	0	2	1	0	1	1
	Ighv1-32P	0	0	0	0	0	0	0	0	0	0	0	0	0	0	0	0	0	0	0	0	0	0	0	0	0	0	0	0	0	0
	Ighv1-31	0	0	0	0	0	2	1	0	1	1	0	0	0	0	0	0	0	5	2	3	0	0	0	0	0	4	0	0	1	2
	Ighv1-26	0	0	0	0	0	2	6	4	4	2	0	0	0	0	0	6	9	2	6	4	0	0	0	0	0	0	2	0	1	1
	Ighv1-25P	0	0	0	0	0	2	17	15	11	8	0	0	0	0	0	0	11	1	4	6	0	2	7	3	4	9	6	11	9	3
	Ighv1-23 ORF	0	0	0	0	0	1	7	5	4	3	1	1	0	1	1	2	5	4	4	2	0	0	0	0	0	0	5	4	3	3
	Ighv1-22	1	1	0	1	1	4	7	10	7	3	0	0	1	0	1	0	0	1	0	1	0	0	0	0	0	0	2	0	1	1
	Ighv1-21P	0	0	0	0	0	0	7	7	5	4	0	0	0	0	0	0	3	0	1	2	0	0	0	0	0	8	6	0	5	4
J558	Ighv1-21-1P	0	0	1	0	1	0	0	3	1	2	0	0	0	0	0	0	0	0	0	0	0	0	0	0	0	0	3	3	2	2
	Ighv1-20	0	0	1	0	1	0	0	0	0	0	0	1	0	0	1	1	0	0	0	1	0	0	0	0	0	0	0	5	2	3
	Ighv1-19	0	0	0	0	0	4	0	0	1	2	0	0	2	1	1	2	0	7	3	4	0	0	2	1	1	3	3	7	4	2
	Ighv1-18	0	0	1	0	1	7	6	8	7	1	0	0	4	1	2	0	1	2	1	1	1	0	0	0	1	3	4	10	6	4
	Ighv1-17	0	0	0	0	0	0	1	9	3	5	0	0	0	0	0	0	0	0	0	0	0	0	0	0	0	0	0	0	0	0
	Ighv1-15	0	0	0	0	0	2	9	2	4	4	1	0	0	0	1	0	0	3	1	2	0	0	0	0	0	0	0	1	0	1
	Ighv1-14	0	0	1	0	1	0	2	0	1	1	0	0	1	0	1	0	0	1	0	1	0	0	0	0	0	0	2	0	1	1
	Ighv1-13P	0	1	3	1	2	0	6	3	3	3	0	0	0	0	0	0	5	0	2	3	0	0	4	1	2	1	0	0	0	1
	Ighv1-12	0	0	8	3	5	42	33	19	31	12	7	2	0	3	4	20	15	6	14	7	10	4	10	8	3	23	29	25	26	3
	Ighv1-11	14	16	11	14	3	85	43	67	65	21	21	14	21	19	4	39	35	63	46	15	35	53	47	45	9	41	31	66	46	18
	Ighv1-9	4	2	17	8	8	15	12	17	15	3	0	6	4	3	3	10	5	3	6	4	5	6	8	6	2	6	11	18	12	6
	Ighv15-2	7	0	2	3	4	17	12	16	15	3	4	5	3	4	1	1	10	6	6	5	18	14	7	13	6	6	5	18	10	7
	Ighv1-7	1	0	0	0	1	3	9	12	8	5	0	0	7	2	4	1	0	11	4	6	1	3	6	3	3	11	1	0	4	6
	Ighv10-3	0	2	5	2	3	13	7	16	12	5	0	2	2	1	1	1	6	19	9	9	2	5	10	6	4	13	8	7	9	3
	Ighv1-5	0	0	2	1	1	7	0	0	2	4	0	0	1	0	1	4	0	3	2	2	0	0	0	0	0	0	0	6	2	3
	Ighv1-4	0	0	1	0	1	0	0	0	0	0	0	1	0	0	1	1	0	0	0	1	0	4	1	2	2	0	1	1	1	1
	Ighv10-1	3	9	1	4	4	13	30	18	20	9	1	2	5	3	2	7	6	12	8	3	1	15	26	14	13	7	13	31	17	12
	Ighv1-2P	1	2	5	3	2	2	2	1	2	1	0	0	5	2	3	1	1	6	3	3	1	0	1	1	1	1	3	0	1	2
	Ighv8-2 ORF	0	0	3	1	2	3	0	0	1	2	0	0	4	1	2	0	0	7	2	4	0	0	2	1	1	3	0	0	1	2
	Ighv6-7	0	0	1	0	1	0	0	2	1	1	0	1	0	0	1	1	0	0	0	1	0	0	0	0	0	0	0	0	0	0
	Ighv6-6	4	12	1	6	6	23	45	27	32	12	2	4	2	3	1	4	9	11	8	4	2	12	11	8	6	12	15	20	16	4
	Ighv6-5	0	0	2	1	1	3	4	1	3	2	0	0	0	0	0	1	0	6	2	3	1	1	0	1	1	0	4	2	2	2
	Ighv6-4	0	0	0	0	0	1	3	5	3	2	0	0	0	0	0	0	0	2	1	1	0	0	0	0	0	0	1	7	3	4
	Ighv6-3	3	0	0	1	2	11	13	14	13	2	0	7	2	3	4	5	18	13	12	7	10	2	6	6	4	2	12	18	11	8
	Ighv12-3	7	2	26	12	13	50	80	73	68	16	13	9	11	11	2	22	45	36	34	12	11	23	22	19	7	35	45	52	44	9
	Ighv13-2	8	11	6	8	3	30	53	47	43	12	10	16	8	11	4	28	15	27	23	7	37	23	41	34	9	18	30	40	29	11
	Ighv3-8	2	0	7	3	4	48	23	36	36	13	6	8	11	8	3	10	15	20	15	5	9	11	24	15	8	14	14	24	17	6
	Ighv5-21P	0	2	0	1	1	4	0	0	1	2	0	6	0	2	3	0	0	0	0	0	2	2	0	1	1	0	0	0	0	0
	Ighv9-4	0	0	0	0	0	6	11	7	8	3	0	0	5	2	3	0	5	3	3	3	7	7	7	7	0	8	0	5	4	4
	Ighv3-6	1	7	4	4	3	0	14	20	11	10	5	1	1	2	2	5	3	14	7	6	1	15	12	9	7	9	15	12	12	3
	Ighv13-1	0	0	0	0	0	0	0	0	0	0	0	0	0	0	0	0	0	0	0	0	0	0	0	0	0	0	0	0	0	0
	Ighv3-5	0	0	0	0	0	2	0	0	1	1	0	0	0	0	0	1	0	1	1	1	0	2	0	1	1	0	0	9	3	5
	Ighv3-4	1	6	1	3	3	11	14	3	9	6	5	2	2	3	2	3	5	8	5	3	1	5	3	3	2	4	2	13	6	6
	Ighv7-4	0	2	0	1	1	9	2	15	9	7	0	0	0	0	0	0	4	0	1	2	1	0	0	0	1	3	6	0	3	3
	Ighv3-3	1	6	5	4	3	6	23	9	13	9	1	4	12	6	6	9	5	5	6	2	0	14	9	8	7	2	17	28	16	13
	Ighv14-4	0	4	7	4	4	10	12	6	9	3	0	3	5	3	3	4	4	7	5	2	13	6	13	11	4	2	1	6	3	3
Middle	Ighv7-3	3	0	4	2	2	7	8	21	12	8	0	2	1	1	1	2	5	14	7	6	3	10	46	20	23	9	12	11	11	2
	Ighv9-3	0	2	11	4	6	5	11	9	8	3	2	3	9	5	4	0	9	0	3	5	6	9	4	6	3	8	11	8	9	2
	Ighv12-2P	0	0	1	0	1	0	0	0	0	0	0	0	0	0	0	0	0	0	0	0	0	0	0	0	0	0	0	0	0	0
	Ighv9-2	0	0	3	1	2	2	5	3	3	2	0	0	0	0	0	0	10	0	3	6	5	8	1	5	4	0	2	0	1	1
	Ighv12-1	2	10	0	4	5	0	9	2	4	5	2	0	3	2	2	0	1	2	1	1	1	0	0	0	1	2	5	0	2	3
	Ighv9-1	1	0	0	0	1	0	1	2	1	1	1	0	1	1	1	0	0	2	1	1	2	13	2	6	6	2	2	0	1	1
	Ighv16-1	0	0	0	0	0	0	0	0	0	0	0	0	0	0	0	0	0	0	0	0	0	0	0	0	0	0	0	1	0	1
	Ighv14-3	0	0	0	0	0	0	1	13	5	7	3	1	2	2	1	1	6	9	5	4	10	0	3	4	5	11	0	0	4	6
	Ighv11-2	3	1	4	3	2	21	15	32	23	9	15	3	10	9	6	10	3	4	6	4	27	3	33	21	16	8	15	23	15	8
	Ighv3-2	10	1	0	4	6	20	4	9	11	8	3	4	12	6	5	2	9	5	5	4	11	14	16	14	3	4	4	24	11	12
	Ighv4-2	0	0	3	1	2	6	4	3	4	2	2	5	6	4	2	2	4	2	3	1	22	5	9	12	9	3	2	12	6	6
	Ighv14-2	8	1	4	4	4	12	1	9	7	6	17	0	5	7	9	8	5	5	6	2	32	5	24	20	14	1	6	6	4	3
	Ighv11-1	10	8	2	7	4	7	7	11	8	2	12	14	12	13	1	1	3	1	2	1	27	25	45	32	11	12	7	8	9	3
	Ighv3-1	0	0	5	2	3	5	6	7	6	1	1	4	3	3	2	1	0	0	0	1	5	3	6	5	2	0	0	9	3	5
	Ighv4-1	0	3	2	2	2	5	5	0	3	3	4	8	5	6	2	5	2	0	2	3	10	12	20	14	5	4	3	2	3	1
	Ighv14-1	0	0	1	0	1	0	0	3	1	2	0	3	0	1	2	0	0	0	0	0	0	0	3	1	2	0	4	0	1	2
	Ighv7-2	0	0	0	0	0	0	0	0	0	0	0	0	0	0	0	0	0	0	0	0	0	0	0	0	0	0	0	0	0	0
	Ighv7-1	11	17	12	13	3	14	30	7	17	12	21	13	24	19	6	0	10	20	10	10	40	40	58	46	10	20	11	14	15	5
	Ighv2-9	10	8	15	11	4	13	3	5	7	5	38	12	11	20	15	8	11	22	14	7	25	32	48	35	12	22	13	17	17	5

	Ighv5-17	32	10	12	18	12	5	17	8	10	6	23	22	13	19	6	5	4	12	7	4	30	48	66	48	18	3	24	10	12	11
	Ighv5-16	3	3	6	4	2	8	6	8	7	1	3	4	5	4	1	3	7	5	5	2	2	11	12	8	6	2	6	16	8	7
	Ighv5-15	13	9	11	11	2	4	7	5	5	2	21	9	14	15	6	0	8	8	5	5	5	12	27	15	11	2	14	8	8	6
	Ighv2-7	0	0	1	0	1	1	0	0	0	1	0	0	0	0	0	0	0	0	0	0	0	0	0	0	0	0	0	3	1	2
	Ighv2-6-8	7	7	11	8	2	6	9	0	5	5	11	8	12	10	2	6	5	6	6	1	0	5	8	4	4	0	2	16	6	9
	Ighv2-9-1	2	9	7	6	4	3	4	0	2	2	13	35	18	22	12	7	3	8	6	3	15	22	26	21	6	4	3	3	3	1
	Ighv5-12-4	0	0	1	0	1	0	0	0	0	0	0	0	0	0	0	0	0	0	0	0	0	0	1	0	1	0	0	0	0	0
	Ighv5-9-1	4	14	14	11	6	3	5	11	6	4	21	27	32	27	6	5	0	3	3	3	29	58	83	57	27	1	2	1	1	1
**7183/Q52**	Ighv2-6	29	42	18	30	12	2	3	6	4	2	15	35	14	21	12	11	1	1	4	6	36	44	62	47	13	1	1	7	3	3
	Ighv5-12	49	54	46	50	4	11	14	12	12	2	65	92	95	84	17	9	4	3	5	3	128	169	188	162	31	19	20	10	16	6
	Ighv2-5	7	25	18	17	9	4	0	2	2	2	12	19	16	16	4	8	1	2	4	4	26	44	23	31	11	2	12	15	10	7
	Ighv5-9	3	1	8	4	4	4	16	0	7	8	3	0	4	2	2	0	2	5	2	3	0	6	5	4	3	0	0	6	2	3
	Ighv2-4	0	2	2	1	1	0	0	0	0	0	8	2	2	4	3	5	0	0	2	3	2	5	3	3	2	2	1	3	2	1
	**Ighv5-6**	78	62	65	68	9	3	15	12	10	6	86	83	81	83	3	3	4	10	6	4	122	215	262	200	71	26	13	6	15	10
	**Ighv2-3**	96	135	128	120	21	14	22	1	12	11	87	92	73	84	10	13	8	14	12	3	55	161	141	119	56	23	4	11	13	10
	**Ighv5-4**	128	164	149	147	18	9	16	17	14	4	147	172	135	151	19	15	14	15	15	1	185	200	243	209	30	66	17	16	33	29
	**Ighv2-2**	318	442	393	384	62	27	59	36	41	17	296	315	222	278	49	19	13	29	20	8	285	361	419	355	67	77	8	85	57	42
	**Ighv5-2**	866	960	992	939	65	44	74	87	68	22	466	526	477	490	32	40	36	43	40	4	593	746	822	720	117	101	47	81	76	27
	**Ighv5-1P**	35	38	62	45	15	4	16	8	9	6	33	20	16	23	9	0	3	5	3	3	27	15	20	21	6	2	5	2	3	2
	D usage in DJH joins																														
	**IGHD1-1 (DFL16.1)**	75965	80946	78077	78329	2500	5292	5843	5868	5668	326	66716	61828	58299	62281	4227	4715	5485	4728	4976	441	44816	64508	81613	63646	18414	6502	6205	8223	6977	1090
	**IGHD6-1**	343	277	312	311	33	59	46	50	52	7	227	243	199	223	22	30	39	56	42	13	304	371	337	337	34	108	36	62	69	36
	**IGHD2-3**	16495	17329	17930	17251	721	4502	4742	5180	4808	344	13499	19300	14294	15698	3145	4428	3515	3647	3863	493	24403	21368	23121	22964	1524	12387	5664	6816	8289	3595
	**IGHD6-2**	65	56	71	64	8	20	25	21	22	3	41	116	43	67	43	15	1	9	8	7	144	98	106	116	25	52	24	29	35	15
	**IGHD2-4**	3320	3134	3336	3263	112	1109	1146	1273	1176	86	2618	2580	2379	2526	128	867	997	824	896	90	5340	2994	4256	4197	1174	2821	1331	1696	1949	777
	**IGHD2-5**	616	506	499	540	66	245	264	244	251	11	330	358	357	348	16	123	156	136	138	17	818	403	559	593	210	528	187	235	317	185
	**IGHD2-6**	450	390	427	422	30	212	204	173	196	21	312	232	286	277	41	125	106	134	122	14	537	307	378	407	118	351	133	221	235	110
	**IGHD2-7**	348	356	439	381	50	190	148	207	182	30	287	225	182	231	53	110	169	115	131	33	495	181	365	347	158	422	71	144	212	185
	**IGHD2-8**	3678	4022	3850	3850	172	1802	1984	2015	1934	115	2914	2581	2522	2672	211	1315	1501	1221	1346	142	5040	2623	3792	3818	1209	3615	1360	2096	2357	1150
	**IGHD3-2**	357	488	493	446	77	189	357	248	265	85	338	230	239	269	60	140	188	123	150	34	599	416	507	507	92	368	279	317	321	45
	**IGHD4-1 (DQ52)**	1863	2177	2192	2077	186	1922	2067	2049	2013	79	1455	1411	1254	1373	106	999	1091	973	1021	62	1643	1512	1788	1648	138	1349	1159	1571	1360	206
	D usage in VHDJH joins																														
	**IGHD1-1 (DFL16.1)**	743	815	837	798	49	472	516	612	533	71	774	772	624	723	86	256	372	315	314	58	780	1109	1496	1128	358	244	459	577	427	169
	**IGHD6-1**	0	0	6	2	3	1	0	5	2	3	1	3	0	1	2	0	2	2	1	1	1	5	2	3	2	2	2	1	2	1
	**IGHD2-3**	485	575	573	544	52	630	641	626	633	8	387	515	570	491	94	347	291	358	332	36	734	875	963	857	115	528	467	589	528	61
	**IGHD6-2**	0	6	5	4	3	4	0	2	2	2	0	0	0	0	0	0	0	0	0	0	7	1	2	3	4	2	2	5	3	2
	**IGHD2-4**	141	163	164	156	13	116	174	100	130	39	104	108	99	104	5	49	47	81	59	19	177	169	233	193	35	127	62	111	100	34
	**IGHD2-5**	49	60	56	55	5	35	47	30	37	9	26	27	33	29	4	17	16	18	17	1	37	47	46	43	5	29	15	38	27	12
	**IGHD2-6**	49	60	56	55	5	35	47	30	37	9	26	27	33	29	4	17	16	18	17	1	37	47	46	43	5	29	15	38	27	12
	**IGHD2-7**	33	22	58	38	18	8	25	21	18	9	13	19	22	18	5	1	8	10	7	5	44	22	2	23	21	18	5	5	9	8
	**IGHD2-8**	160	186	184	177	15	151	182	143	158	21	76	67	65	69	5	34	46	93	58	31	94	103	138	111	24	71	80	118	90	25
	**IGHD3-2**	19	32	39	30	10	12	17	30	20	10	11	12	9	10	2	7	5	14	9	5	16	41	21	26	13	9	14	13	12	2
	**IGHD4-1 (DQ52)**	45	60	44	50	9	22	28	58	36	19	24	23	9	18	8	9	17	33	20	12	11	35	54	33	22	13	8	32	18	12
	D usage in DJH+VHDJH joins																														
	**IGHD1-1 (DFL16.1)**	76708	81761	78914	79128	2534	5764	6359	6480	6201	383	67490	62600	58923	63004	4297	4971	5857	5043	5290	492	45596	65617	83109	64774	18771	6746	6664	8800	7403	1210
	**IGHD6-1**	343	277	318	313	33	60	46	55	54	7	228	246	199	224	24	30	41	58	43	14	305	376	339	340	35	110	38	63	70	37
	**IGHD2-3**	16980	17904	18503	17796	768	5132	5383	5806	5441	340	13886	19815	14864	16188	3179	4775	3806	4005	4195	512	25137	22243	24084	23821	1465	12915	6131	7405	8817	3606
	**IGHD6-2**	65	62	76	68	7	24	25	23	24	1	41	116	43	67	43	15	1	9	8	7	151	99	108	119	28	54	26	34	38	15
	**IGHD2-4**	3461	3297	3500	3419	108	1225	1320	1373	1306	75	2722	2688	2478	2629	133	916	1044	905	955	77	5517	3163	4489	4390	1180	2948	1393	1807	2049	806
	**IGHD2-5**	665	566	555	595	61	280	311	274	288	20	356	385	390	377	19	140	172	154	155	16	855	450	605	637	204	557	202	273	344	188
	**IGHD2-6**	499	450	483	477	25	247	251	203	234	27	338	259	319	306	41	142	122	152	139	15	574	354	424	451	112	380	148	259	262	116
	**IGHD2-7**	381	378	497	419	67	198	173	228	200	27	300	244	204	249	48	111	177	125	138	35	539	203	367	370	168	440	76	149	222	193
	**IGHD2-8**	3838	4208	4034	4027	185	1953	2166	2158	2092	121	2990	2648	2587	2742	217	1349	1547	1314	1403	126	5134	2726	3930	3930	1204	3686	1440	2214	2447	1141
	**IGHD3-2**	376	520	532	476	87	201	374	278	284	87	349	242	248	279	60	147	193	137	159	30	615	457	528	533	79	377	293	330	333	42
	**IGHD4-1 (DQ52)**	1908	2237	2236	2127	190	1944	2095	2107	2049	91	1479	1434	1263	1392	114	1008	1108	1006	1041	58	1654	1547	1842	1681	150	1362	1167	1603	1377	218

Notes:
1. Data were nomalized to 1,964,102 total reads.
2. Proximal V and D segments are denoted in bold.

Supplementary Table 4 | Absolute utilization of Igh D to JHs1-4 in WAPL-degron v-Abl pro-B cells. Data was extracted from previously published data (22: GSM4593296-GSM4593303)

	WT untreated JH1 bait primer (n=3)					WT untreated JH2 bait primer (n=3)					WT untreated JH3 bait primer (n=3)					WT untreated JH4 bait primer (n=3)				
	#1	#2	#3	Average	s.d.	#1	#2	#3	Average	s.d.	#1	#2	#3	Average	s.d.	#1	#2	#3	Average	s.d.
D usage in DJH joins																				
**IGHD1-1 (DFL16.1)**	**108484**	**113218**	**96778**	**106160**	**8463**	**173771**	**182460**	**164495**	**173575**	**8984**	**46828**	**53634**	**47131**	**49198**	**3845**	**23338**	**23810**	**20867**	**22672**	**1581**
**IGHD6-1**	**104**	**89**	**85**	**93**	**10**	**736**	**555**	**553**	**615**	**105**	**195**	**163**	**137**	**165**	**29**	**63**	**45**	**27**	**45**	**18**
**IGHD2-3**	**6500**	**6020**	**4998**	**5839**	**767**	**13214**	**13239**	**10743**	**12399**	**1434**	**4732**	**4528**	**3744**	**4335**	**522**	**2510**	**2244**	**1834**	**2196**	**341**
**IGHD6-2**	**12**	**22**	**15**	**16**	**5**	**73**	**72**	**70**	**72**	**2**	**16**	**27**	**21**	**21**	**6**	**7**	**3**	**11**	**7**	**4**
**IGHD2-4**	**1749**	**1662**	**1562**	**1658**	**94**	**4546**	**4121**	**3897**	**4188**	**330**	**1456**	**1456**	**1288**	**1400**	**97**	**669**	**582**	**483**	**578**	**93**
**IGHD2-5**	**522**	**445**	**427**	**465**	**50**	**1140**	**1010**	**1066**	**1072**	**65**	**292**	**278**	**239**	**270**	**27**	**173**	**106**	**119**	**133**	**36**
**IGHD2-6**	**425**	**352**	**346**	**374**	**44**	**839**	**766**	**791**	**799**	**37**	**205**	**182**	**180**	**189**	**14**	**114**	**100**	**100**	**105**	**8**
**IGHD2-7**	**181**	**216**	**195**	**197**	**18**	**595**	**624**	**506**	**575**	**61**	**158**	**159**	**122**	**146**	**21**	**60**	**71**	**40**	**57**	**16**
**IGHD2-8**	**2743**	**2782**	**2107**	**2544**	**379**	**5239**	**4995**	**4562**	**4932**	**343**	**1386**	**1399**	**1236**	**1340**	**91**	**816**	**757**	**534**	**702**	**149**
**IGHD3-2**	**135**	**131**	**114**	**127**	**11**	**1702**	**1603**	**1335**	**1547**	**190**	**426**	**422**	**316**	**388**	**62**	**124**	**101**	**104**	**110**	**13**
**IGHD4-1 (DQ52)**	**12801**	**12213**	**9782**	**11599**	**1601**	**48201**	**44001**	**38932**	**43711**	**4641**	**7441**	**7342**	**5820**	**6868**	**909**	**1342**	**1135**	**877**	**1118**	**233**

	WT+Dox/IAA JH1 bait primer (n=3)					WT+Dox/IAA JH2 bait primer (n=3)					WT+Dox/IAA JH3 bait primer (n=3)					WT+Dox/IAA JH4 bait primer (n=3)				
	#1	#2	#3	Average	s.d.	#1	#2	#3	Average	s.d.	#1	#2	#3	Average	s.d.	#1	#2	#3	Average	s.d.
D usage in DJH joins																				
**IGHD1-1 (DFL16.1)**	**5362**	**5383**	**5609**	**5451**	**137**	**8355**	**7916**	**8341**	**8204**	**250**	**1857**	**1814**	**1796**	**1822**	**31**	**789**	**816**	**899**	**835**	**57**
**IGHD6-1**	**11**	**7**	**13**	**10**	**3**	**79**	**66**	**58**	**68**	**11**	**9**	**9**	**10**	**9**	**1**	**4**	**1**	**6**	**4**	**3**
**IGHD2-3**	**1051**	**1042**	**1215**	**1103**	**97**	**2053**	**1973**	**2036**	**2021**	**42**	**595**	**592**	**581**	**589**	**7**	**336**	**366**	**387**	**363**	**26**
**IGHD6-2**	**1**	**0**	**2**	**1**	**1**	**15**	**11**	**23**	**16**	**6**	**3**	**1**	**1**	**2**	**1**	**2**	**1**	**0**	**1**	**1**
**IGHD2-4**	**414**	**411**	**402**	**409**	**6**	**852**	**782**	**854**	**829**	**41**	**228**	**225**	**261**	**238**	**20**	**132**	**129**	**123**	**128**	**5**
**IGHD2-5**	**122**	**103**	**139**	**121**	**18**	**225**	**202**	**215**	**214**	**12**	**68**	**46**	**50**	**55**	**12**	**27**	**21**	**36**	**28**	**8**
**IGHD2-6**	**86**	**98**	**123**	**102**	**19**	**165**	**161**	**182**	**169**	**11**	**55**	**28**	**34**	**39**	**14**	**45**	**27**	**36**	**36**	**9**
**IGHD2-7**	**80**	**87**	**76**	**81**	**6**	**144**	**154**	**155**	**151**	**6**	**34**	**21**	**29**	**28**	**7**	**24**	**22**	**16**	**21**	**4**
**IGHD2-8**	**784**	**716**	**904**	**801**	**95**	**1332**	**1263**	**1268**	**1288**	**38**	**342**	**321**	**320**	**328**	**12**	**165**	**185**	**229**	**193**	**33**
**IGHD3-2**	**23**	**29**	**20**	**24**	**5**	**324**	**299**	**291**	**305**	**17**	**75**	**79**	**80**	**78**	**3**	**36**	**34**	**34**	**35**	**1**
**IGHD4-1 (DQ52)**	**7214**	**7232**	**7914**	**7453**	**399**	**32811**	**31355**	**33050**	**32405**	**917**	**4880**	**4831**	**5007**	**4906**	**91**	**336**	**348**	**390**	**358**	**28**

Notes:
1. Data using different bait primer were nomalized to the indicated total reads: 672.689 reads for JH1 primer: 946.994 reads for JH2 primer: 410.987 reads for JH3 primer: 614.285 reads for JH4 primer.
2. Data was extracted from previously published data (22: GSM4593296-GSM4593303)

**Supplementary Table 5 | Oligos used in this study.**	
**Genotyping primers**	
CBE1 WT Forward	AGTCTCTGCTTCCCCCTTGT
CBE1 WT Reverse	AGCACATGACCCTTCTCACC
CBE1 scramble Forward	AGCACATGACCCTTCTCACC
CBE1 scramble Reverse	CTCTCCTGCTAGCCTTCTCG
CBE1 inversion Forward	CAGTAATGCTCATGGCCACAAG
CBE1 inversion Reverse	AGCACATGACCCTTCTCACC
CBE2 WT Forward	TCTCCACAAGAGGGCAGAAT
CBE2 WT Reverse	CTTATGGAATGGGTGGGTTG
CBE2 scramble Forward	CTTATGGAATGGGTGGGTTG
CBE2 scramble Reverse	GGGAGATCTGTATCTCTTCCACTAGTAA
CBE2 inversion Forward	ATCTCCACAAGAGGGCAGAAG
CBE2 inversion Reverse	TGTCACCCTTGAGGCCATTC
**sgRNA used for 3'CBE deletion and inversion**	
3'CBE sgRNA-1	AGCCAGTGTTATAGTATCTC
3'CBE sgRNA-2	AGTCCCAACTTTTGCTTTAG
3'CBE sgRNA-3	GTCCCAACTTTTGCTTTAGC
**Oligos for generating HTGTS-V(D)J-seq and 3C-HTGTS libraries**	
**HTGTS-VDJ-seq bait primers**	
JH4 Coding end bio primer (129SV background)	/5BiosG/CCCTCAGGGACAAATATCCA
JH4 Coding end nested primer (129SV background)	CTGCAATGCTCAGAAAACTCC
JH4 Coding end bio primer (C57B6/L background)	/5BiosG/CTGCAATGCTCAGAAAACTCC
JH4 Coding end nested primer (C57B6/L background)	CTTACCTGAGGAGACGGTGAC
**3C-HTGTS bait primers**	
iEμ Bio primer	/5BiosG/GGTTATGTAAGAAATTGAAGGACTTTAGTG
iEμ Nested primer	CTCTATTATTCTTCCCTCTGATTATTGG
VH81X Bio primer	/5BiosG/AAATAGAAGATGAAATGGAAGATTTGAAGG
VH81X Nested primer	TGAGAAACACCAATATTGTCAACTAACC
CBE1 Bio primer	/5BiosG/CATGAGGCTCCTGGAGGTGAC
CBE1 Nested primer	GGTGACCAGGAAAGCTGCTTAG
